# *Myosin7a* Deficiency Results in Reduced Retinal Activity Which Is Improved by Gene Therapy

**DOI:** 10.1371/journal.pone.0072027

**Published:** 2013-08-26

**Authors:** Pasqualina Colella, Andrea Sommella, Elena Marrocco, Umberto Di Vicino, Elena Polishchuk, Marina Garcia Garrido, Mathias W. Seeliger, Roman Polishchuk, Alberto Auricchio

**Affiliations:** 1 Telethon Institute of Genetics and Medicine (TIGEM), Naples, Italy; 2 Division of Ocular Neurodegeneration, Institute for Ophthalmic Research, Centre for Ophthalmology, Tuebingen, Germany; 3 Medical Genetics, Department of Medical Translational Sciences, University of Naples Federico II, Naples, Italy; Justus-Liebig-University Giessen, Germany

## Abstract

Mutations in *MYO7A* cause autosomal recessive Usher syndrome type IB (USH1B), one of the most frequent conditions that combine severe congenital hearing impairment and retinitis pigmentosa. A promising therapeutic strategy for retinitis pigmentosa is gene therapy, however its pre-clinical development is limited by the mild retinal phenotype of the *shaker1* (*sh1^−/−^*) murine model of USH1B which lacks both retinal functional abnormalities and degeneration. Here we report a significant, early-onset delay of *sh1^−/−^* photoreceptor ability to recover from light desensitization as well as a progressive reduction of both b-wave electroretinogram amplitude and light sensitivity, in the absence of significant loss of photoreceptors up to 12 months of age. We additionally show that subretinal delivery to the *sh1^−/−^* retina of AAV vectors encoding the large MYO7A protein results in significant improvement of *sh1^−/−^* photoreceptor and retinal pigment epithelium ultrastructural anomalies which is associated with improvement of recovery from light desensitization. These findings provide new tools to evaluate the efficacy of experimental therapies for USH1B. In addition, although AAV vectors expressing large genes might have limited clinical applications due to their genome heterogeneity, our data show that AAV-mediated *MYO7A* gene transfer to the *sh1^−/−^* retina is effective.

## Introduction

Usher syndrome (USH) is the most common deafness-blindness disorder inherited as autosomal recessive and due to mutations in one of 12 different genes [1]. Mutations in *MYO7A* that encodes for the unconventional Myosin7a, cause Usher syndrome type IB (USH1B, [MIM 276900]) which accounts for about 50% of USH1 cases [1]. USH1B is one of the most severe forms of USH that leads to severe congenital hearing impairment and progressive vision loss due to retinitis pigmentosa that manifests pre-pubertally [1]. The hearing dysfunction of USH1B patients can be ameliorated by cochlear implants while retinitis pigmentosa is currently untreatable [1]. *MYO7A* gene replacement may represent a valuable therapeutic strategy for the USH1B retina [2], but its development is hampered by difficulties to find an appropriate animal model. Although various rodent models of USH1B that bear mutations in the *Myo7a* gene are available and present with clear hearing and vestibular defects [3], [4], [5], [6], none presents with frank retinal dysfunction and degeneration [7], [8], [9], [10]. Among them, the most studied is the *shaker1^4626SB/4626SB^* mouse (referred to as *sh1^−/−^*), which is homozygous for an early nonsense mutation in the *Myo7a* gene. *Myo7a* encodes for a large 2215 aminoacid actin-based motor protein that is expressed in the cochlear hair cells, retinal photoreceptors (PRs: rods and cones) and retinal pigment epithelium (RPE) [11], [12], [13]. In rods, Myo7a localizes to the connecting cilium where it cooperates to the transport of Rhodopsin (the rod photopigment) to the rod outer segment (OS) discs [14], [15]. In RPE, Myo7a mainly resides at the apical side [11], [13] and it is required for both proper localization of melanosomes to the RPE microvilli (which surround the PR OS) [16], [17] and phagosome motility [18]. Indeed *sh1^−/−^* mice show: i. slower transport of Rhodopsin that accumulates at the PR connecting cilium [14]; ii. slower distal migration of PR OSs [14]; iii. mislocalization of melanosomes, that do not enter into the RPE microvilli [16]; and iv. slower phagosome motility that results in slower OS digestion [18]. Notably, abnormal melanosome motility has also been reported in human Myo7a-deficient RPE cell cultures [13]. However, differently from patients which experience severe electroretinogram (ERG) decline and PR degeneration [19], [20], *sh1^−/−^* mice do not present neither PR cell loss up to 24 months of age nor important ERG reduction, similarly to the other USH1B models [7], [8]. The base for these interspecies differences remained unknown for long time, however it has been recently attributed to the lack in rodents but not in humans of calyceal processes which are the specific photoreceptor structures where USH1 proteins, including MYO7A, localize [21]. Based on the lack of a frank retinal degeneration in *sh1^−/−^* mice, rescue of the ultrastructural PR and RPE defects represents, to date, the only useful outcome measure for experimental therapies tested in the *sh1^−/−^* retina [2]. However, to develop effective and clinically-relevant therapeutic strategies for the USH1B retina, it is critical to establish if these ameliorate retinal function in *Myo7a-*deficient mice. In the present study we identified significant, early-onset electrophysiological abnormalities of the *sh1^−/−^* retina that remain stable up to 12 months of age and that are independent of PR degeneration. In addition we show that these abnormalities can be reliably used to assess the efficiency of *MYO7A* gene transfer to the *sh1^−/−^* retina.

## Materials and Methods

### Statistical Analysis

**Figure 1:**
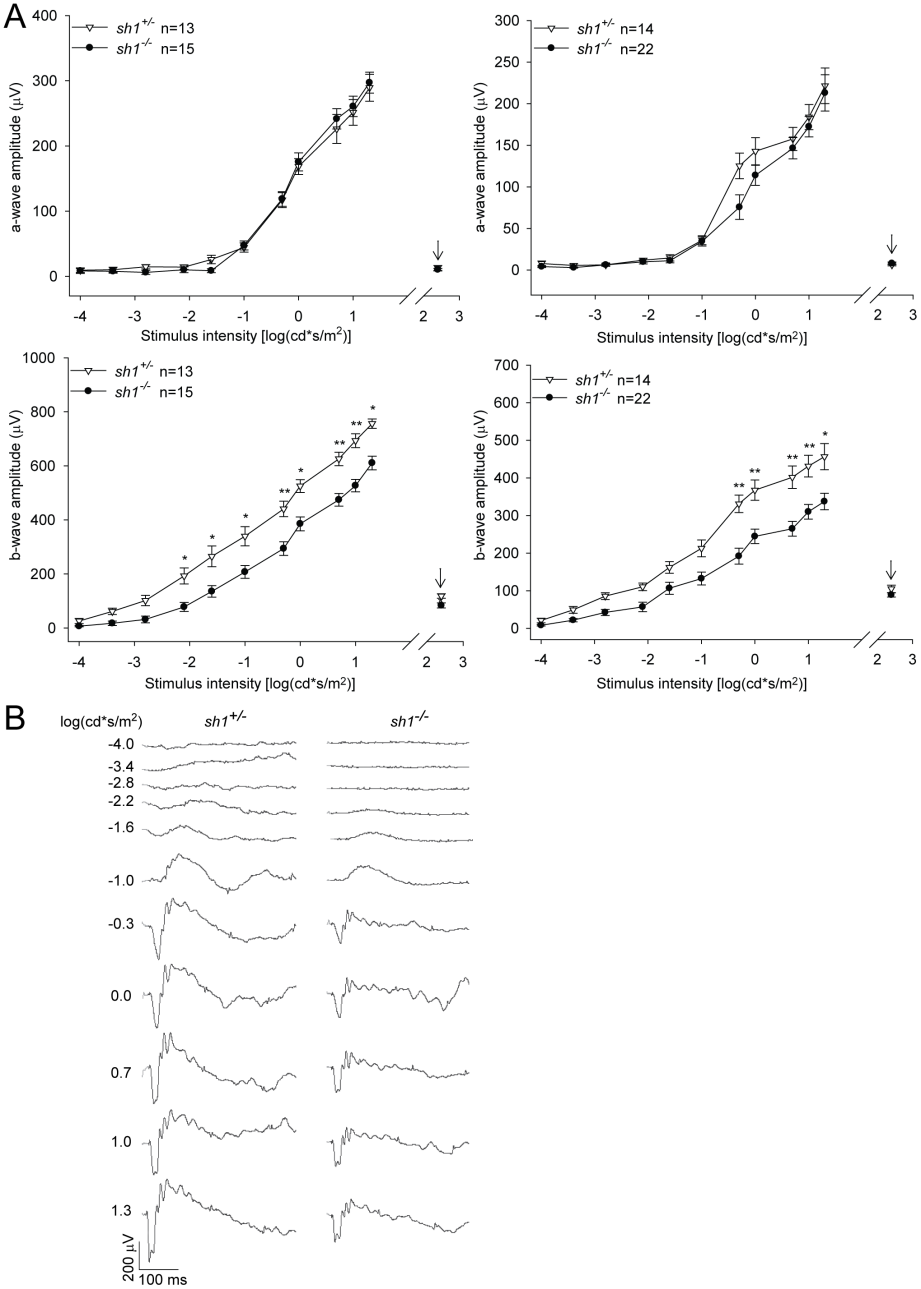
Ganzfeld electroretinograms in albino *sh1* mice. **A.** Mean a- (top panels) and b-(bottom panels) waves from 6-(left panels) and 12-(right panels) month-old *sh1* mice. Data are presented as mean±SEM, n indicates the number of eyes analyzed, the arrows point at the photopic ERG. The statistical significant differences between *sh1^+/−^* and *sh1^−/−^* mice at each specific luminance are marked by asterisks*:* *p value<0.05, **p value<0.001. More details on the statistical analysis including specific statistical values can be found in the Statistical analysis paragraph of the Materials and Methods section. **B.** Representative scotopic ERG waves from one *sh1^+/−^* and one *sh1^−/−^* mouse at 12 months of age**.**

**Figure 2:**
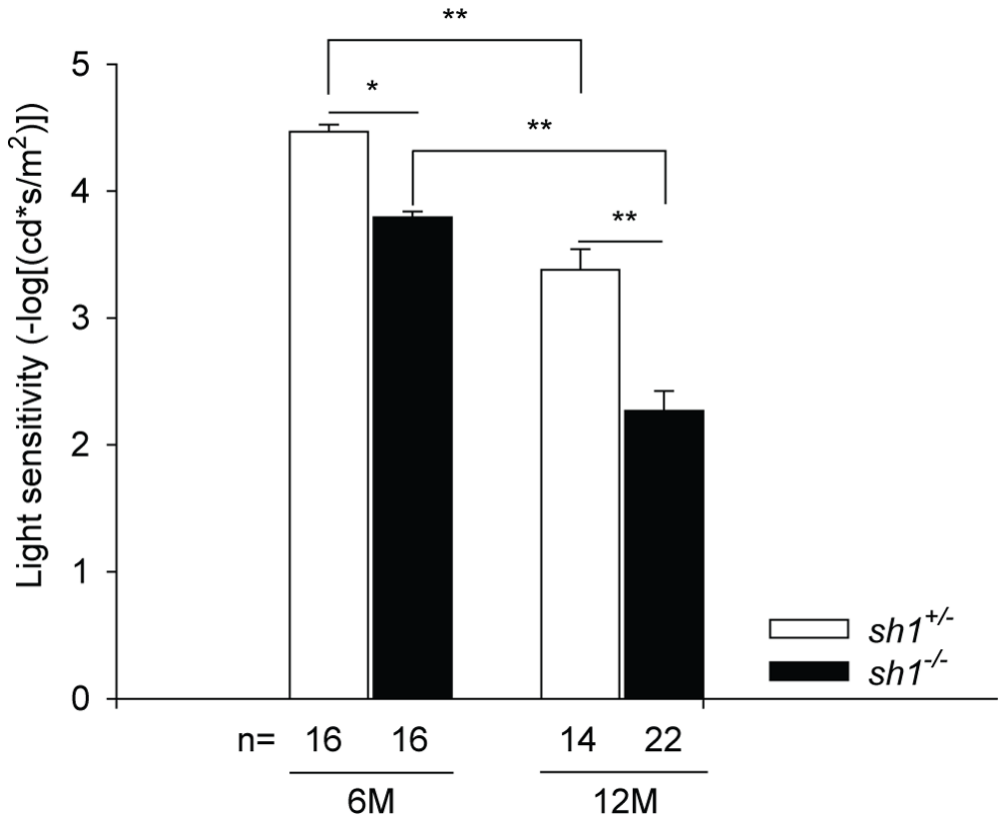
Retinal light sensitivity of albino *sh1* mice . The minimum light intensity (inverted log scale) at which a b-wave shaped response is elicited is shown. Data are presented as mean±SEM, n indicates the number of eyes analyzed, M: months. *p value<0.05, **p value<0.001. More details on the statistical analysis including specific statistical values can be found in the Statistical analysis paragraph of the Materials and Methods section.

**Figure 3:**
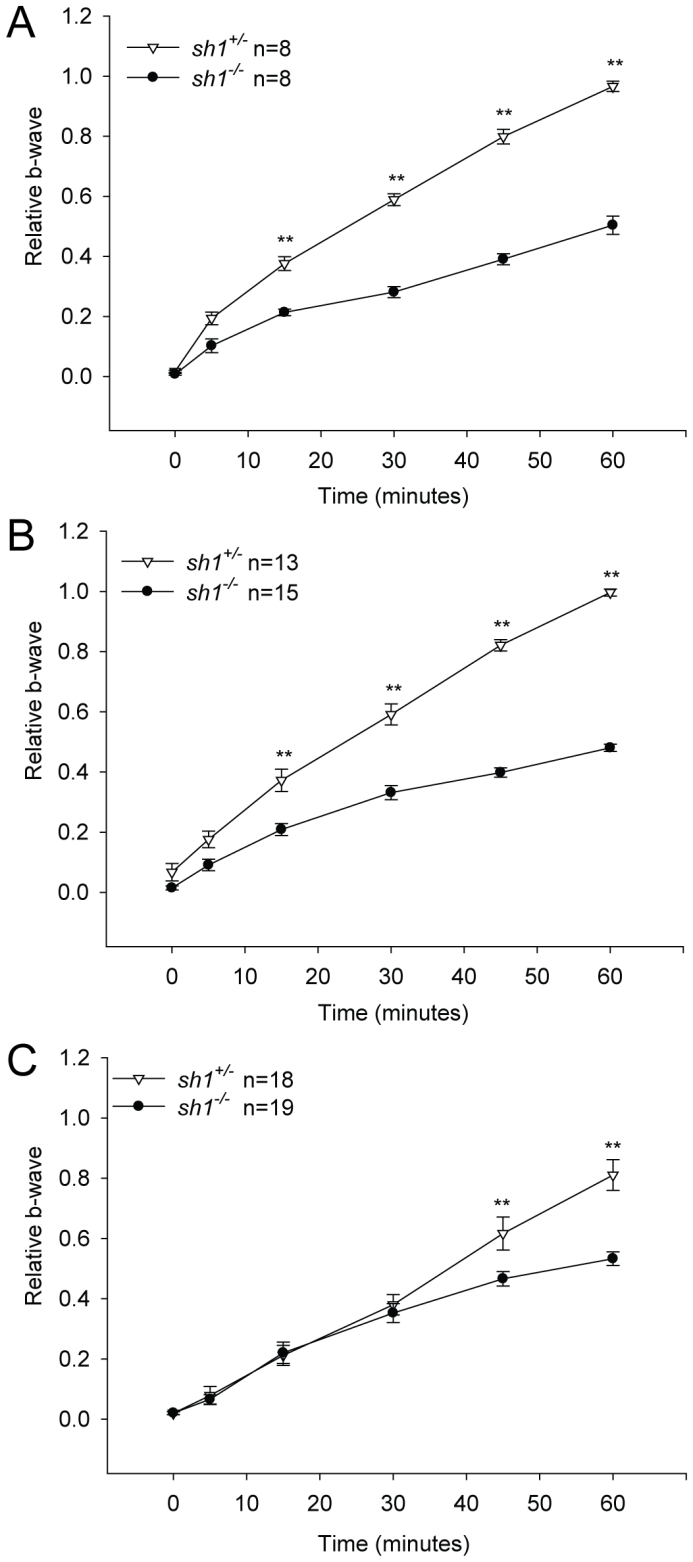
Retinal recovery from light desensitization in albino *sh1* mice . The relative b-wave is the ratio between the post- and the pre-desensitization b-wave amplitudes (µV) both evoked by 1 cd*s/m^2^. The time (minutes) refers to the time post-desensitization. Data are from *sh1* mice at 2 (**A**), 6 (**B**) and 12 (**C**) months of age. Data are presented as mean±SEM; n indicates the number of eyes analyzed. To assess statistical significance Gaussian Processes Bayes Factor (BF) and ANOVA p value were calculated: BF>>1 (**A–C**), p<<2×10^−16^ (**A–B**), p = 5.3×10^−6^ (**C**). The statistical significant differences between *sh1^+/−^* and *sh1^−/−^* mice at each specific time are marked by asterisks*:* **p value<0.05, **p value<0.001. More details on the statistical analysis including specific statistical values can be found in the Statistical analysis paragraph of the Materials and Methods section.

**Figure 4:**
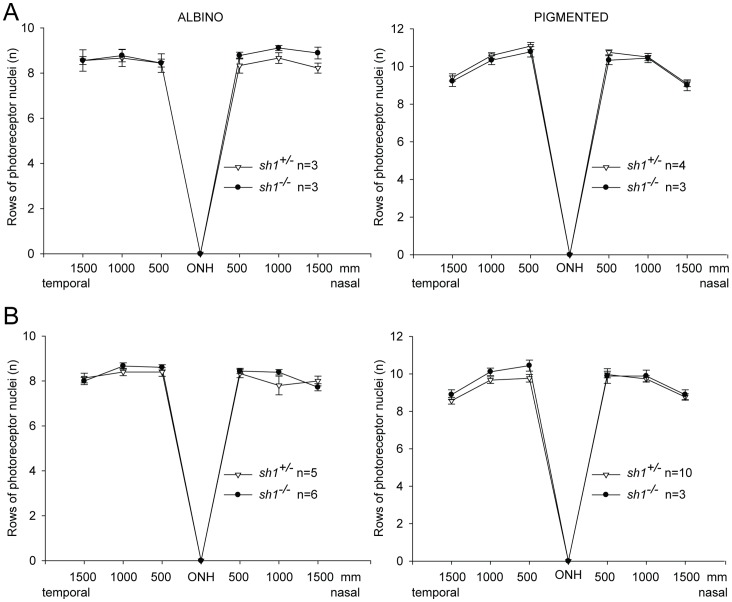
Number of rows of photoreceptor nuclei in the outer nuclear layer of albino (left panels) and pigmented (right panels) *sh1* mice. Data are from mice at 6 (**A**, left panel), 6–7 (**A**, right panel) or 12 (**B**) months of age. Data are presented as mean±SEM; n indicates the number of eyes analyzed; ONH: optic nerve head. No statistically significant differences were found between *sh1^+/−^* and *sh1^−/−^* mice. More details on the statistical can be found in the Statistical analysis paragraph of the Materials and Methods section.

**Figure 5:**
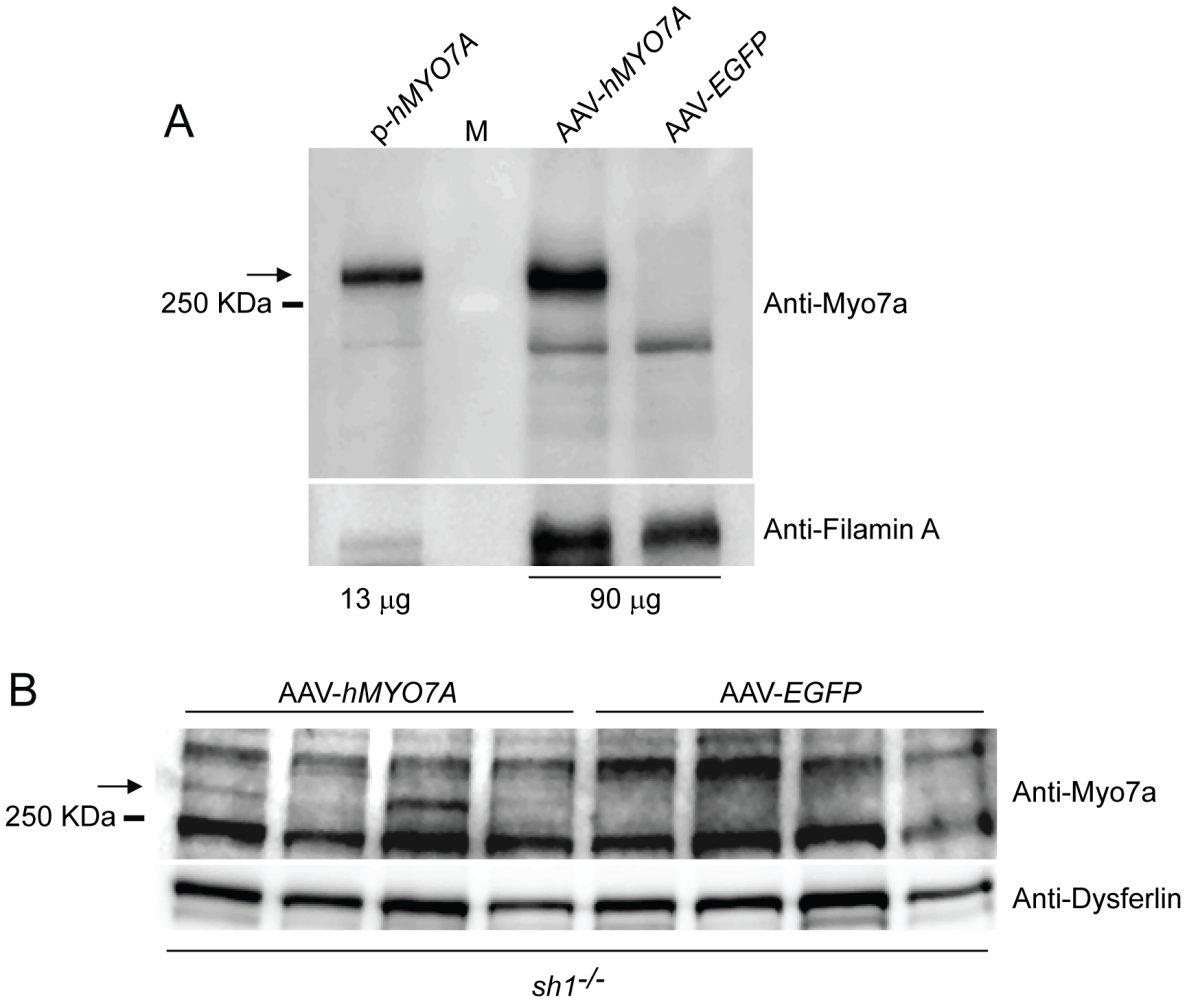
AAV-mediated expression of human Myo7a *in vitro* and in *sh1^−/−^* eyecups. **A**. Western blot analysis on HEK293 cell lysates following infection with AAV2/2-CMV-*hMYO7A* (AAV-*hMYO7A*) or AAV2/2-CMV-*EGFP* (AAV-*EGFP*) or transfection with pAAV2.1-CMV-*hMYO7A* (p*-hMYO7A*). The amount of protein loaded (µg) is showed. The arrow points at the hMyo7a protein. The lysate from HEK293 cells transfected with the pAAV2.1-CMV-*hMYO7A* plasmid (p-*hMYO7A*) was used as positive control. **B**. Western blot analysis of lysate eyecups from *sh1^−/−^* mice 15 months following subretinal injection of AAVs. The arrow points at the hMyo7a protein that was detected in 3/4 *sh1^−/−^* eyecups injected with AAV2/5-CMV*-hMYO7A* (AAV-*hMYO7A*) but not in those injected with AAV2/5-CMV-*EGFP* (AAV-*EGFP,* n = 0/4). One hundred µg of each eyecup lysate were loaded.

**Figure 6:**
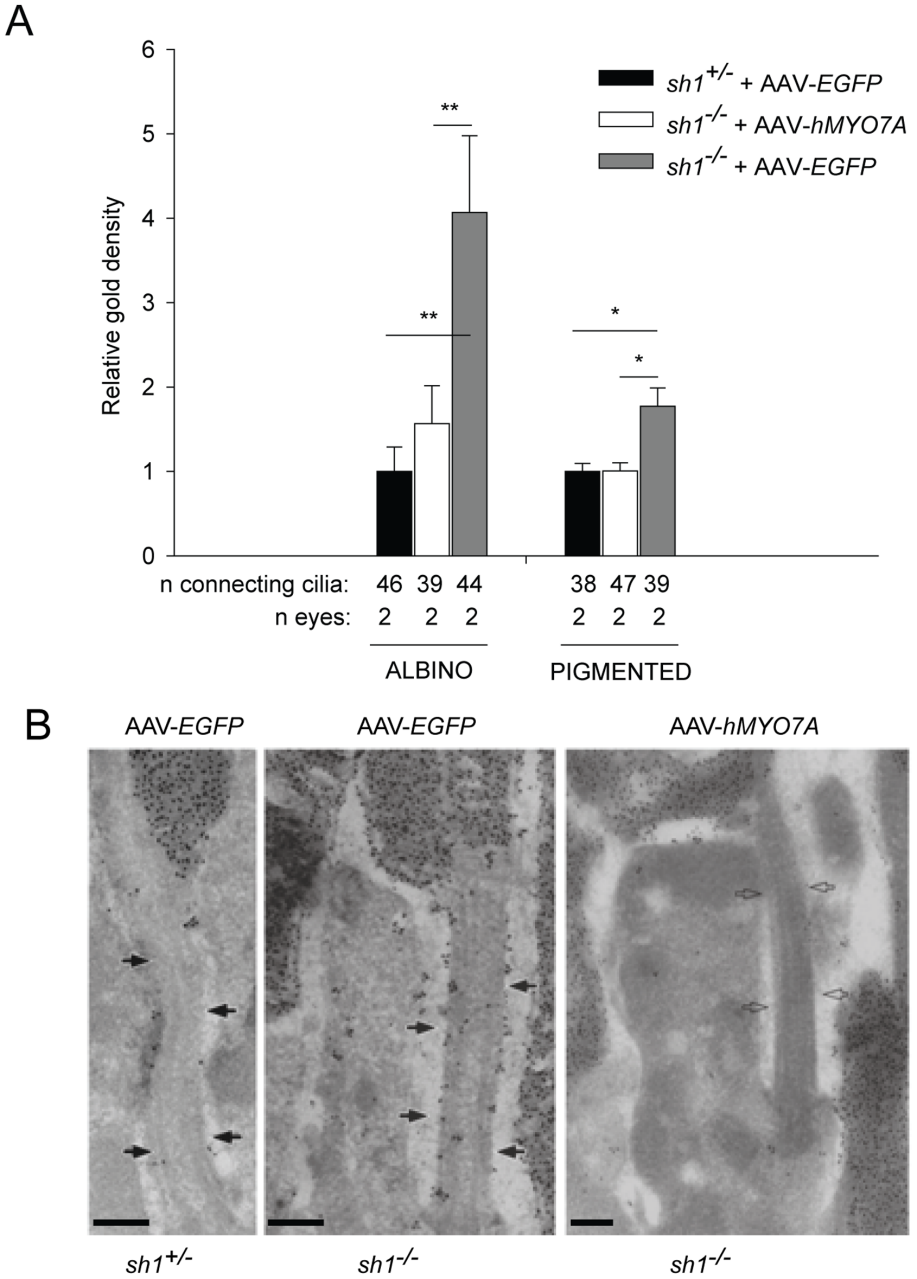
Subretinal delivery of AAV encoding human Myo7a rescues Rhodopsin accumulation in *sh1^−/−^* photoreceptor connecting cilium. **A**. Quantification of the gold particle density (corresponding to Rhodopsin molecule density) in the connecting cilium of *sh1* mice. The quantification is depicted as relative gold density (average gold particle density in *sh1*/average gold particle density in *sh1^+/−^* mice). Albino *sh1* mice were analyzed at 4 months following the injections; pigmented *sh1* mice were analyzed at 4 or 7 months following the injections. Data are presented as mean±SEM. *p value<0.05; **p value<0.001. More details on the statistical analysis including specific statistical values can be found in the Statistical analysis paragraph of the Materials and Methods section. **B**. Representative pictures of photoreceptor connecting cilium containing Rhodopsin immuno-labelled with anti-Rhodopsin antibody in pigmented *sh1* mice at 4 months following the treatment. The black arrows point at the connecting cilium of *sh1^+/−^* and *sh1^−/−^* mice treated with AAV-*EGFP*, while white arrows point at the connecting cilium of *sh1^−/−^* mice treated with AAV-*hMYO7A*. The scale bar is 200 nm. AAV-*hMYO7A*: AAV2/5-CMV-*hMYO7A*; AAV-*EGFP:* AAV2/5-CMV-*EGFP.*

**Figure 7:**
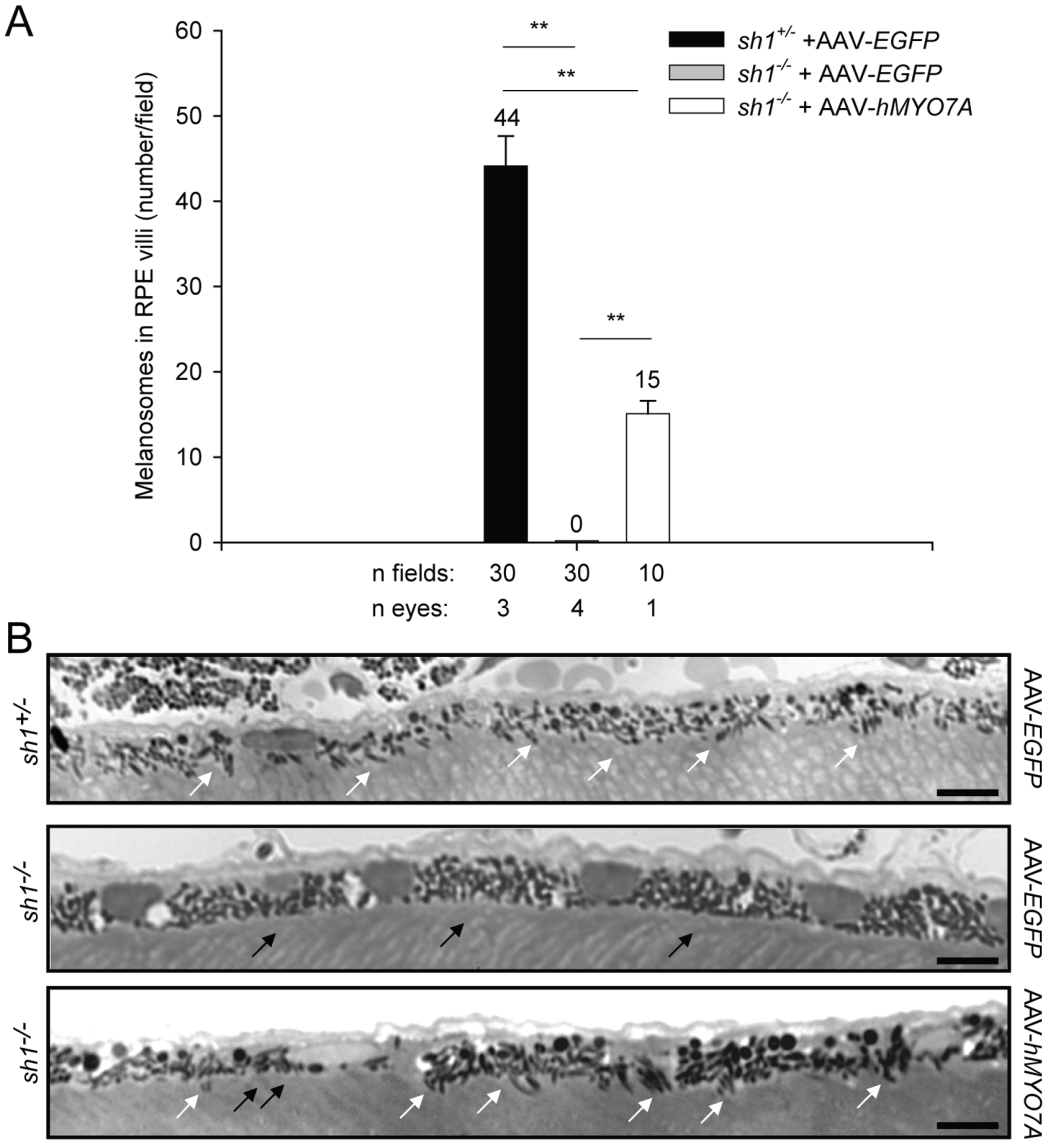
Subretinal delivery of AAV encoding human Myo7a rescues melanosome localization in *sh1^−/−^* RPE villi. **A** ****. Quantification of melanosome localization to the RPE villi of *sh1* mice at 4 months following the treatment. The quantification is depicted as the mean number of melanosomes/field. **p value<0.001. More details on the statistical analysis including specific statistical values can be found in the Statistical analysis paragraph of the Materials and Methods section. **B**. Representative pictures of semi-thin sections stained with Epoxy tissue stain from pigmented *sh1* mice at 4 months following the injections. White arrows point at melanosomes correctly localized to the RPE villi, while black arrows point at mis-localized apical melanosomes. The scale bar is 100 µm. **A–B:** AAV-*hMYO7A*: AAV2/5-CMV-*hMYO7A*; AAV-*EGFP:* AAV2/5-CMV-*EGFP.*

**Figure 8:**
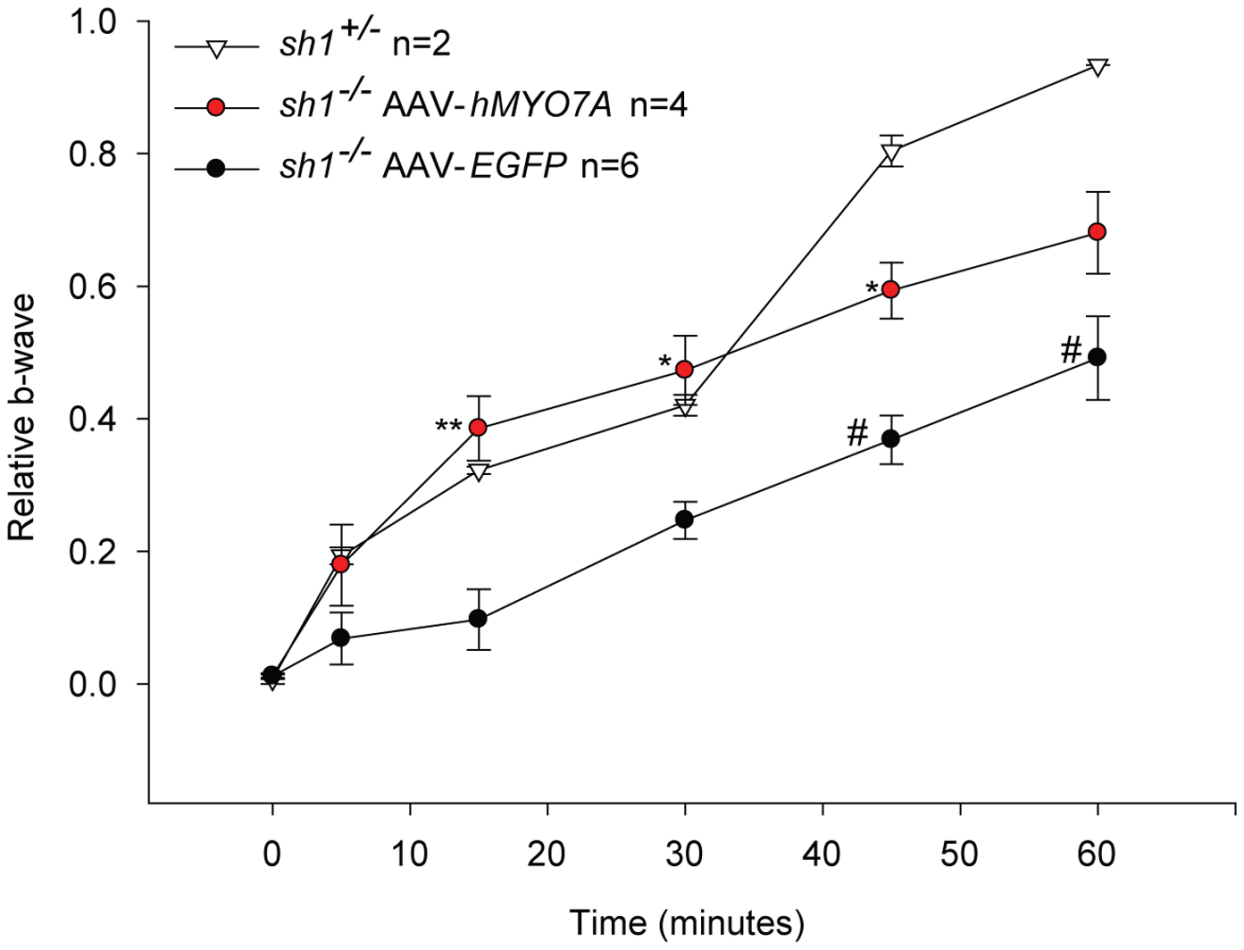
Subretinal delivery of AAV encoding human Myo7a improves recovery from light desensitization in adult *sh1^−/−^* mice. The graph shows the recovery from light desensitization in albino *sh1^−/−^* mice 6 months following AAV injection. The relative b-wave is the ratio between the post- and the pre-desensitization b-wave amplitudes (µV) both evoked by 1 cd*s/m^2^. The time (minutes) refers to the time post-desensitization. To assess statistical significance Two-way ANOVA was used: the statistical significant differences between *sh1^+/−^* and *sh1^−/−^* mice from the post-hoc Multiple Comparison Procedure are depicted: *p value<0.05; ** and # p<0.001. Asterisks label significant differences between *hMYOA*- and *EGFP*-treated eyes; pound keys label significant differences between *EGFP*-treated eyes and *sh1^+/−^* eyes. More details on the statistical analysis can be found in the Statistical analysis paragraph of the Materials and Methods section. AAV-*hMYO7A*: AAV2/5-CMV-*hMYO7A*; AAV-*EGFP:* AAV2/5-CMV-*EGFP.*

Data are presented as mean± standard error of the mean (SEM). Statistical p values<0.05 were considered significant. Two-way ANOVA (factors: genotype and luminance) with post-hoc Multiple Comparison Procedure was used to compare the ERGs recorded from *sh1^+/−^* vs *sh1^−/−^* mice (**Fig. 1A**, **S1A and S2A**). The interaction p values of the ANOVA are the following: **Figure 1A**: p = 0.98 (a-wave 6 months), p = 0.5 (a-wave 12 months), p = 0.002 (b-wave 6 months), p = 8.7×10^−5^ (b-wave 12 months); **Figure S1A**: p = 0.009 (a-wave 3 months), p = 0.96 (b-wave 3 months); **Figure S2A**: p = 0.21 (a-wave 6 months), p = 0.12 (a-wave 12 months), p = 0.09 (b-wave 6 months), p = 0.9 (b-wave 12 months). The statistical significant differences between *sh1^+/−^* and *sh1^−/−^* eyes at each specific luminance were determined with the post-hoc Multiple Comparison Procedure and marked by asterisks in **Figures 1A**, **S1A and S2A.** Two-way ANOVA (factors: age and genotype) with post-hoc Multiple Comparison Procedure was used to compare light sensitivity of *sh1^+/−^* and *sh1^−/−^* mice (**Fig. 2**). The p values of the ANOVA are the following: genotype p = 1.5×10^−10^ **,** age p<<2×10^−16^. The significant comparisons determined with the post-hoc Multiple Comparison Procedure were marked by asterisks in **Figure 2**. The time-course profile of retinal recovery from light desensitization was compared in *sh1^+/−^* vs *sh1^−/−^* mice (**Fig. 3**) by means of two procedures: Gaussian Processes [22] and Two-way ANOVA with post-hoc Multiple Comparison Procedure. Gaussian Processes enable to quantify the true signal and noise embedded in data profiles over time. In particular, given the observed profiles of recovery from light desensitization, two different hypotheses H1 and H2 were compared: H_1_: the recovery profiles are truly differential; H_2_: the difference in recovery profiles is just the effect of random noise. The plausibility of the two different hypotheses is assessed in terms of Bayes Factor (BF) = Probability (Data|H_1_)/Probability (Data|H_2_). The Gaussian Processes Bayes Factors (BF) relative to the recovery profiles depicted in **Figure 3** are the following: 85.06 (**A**), 112.4 (**B**), 17.6 (**C**). The retinal recovery from light desensitization was also compared in *sh1^+/−^* vs *sh1^−/−^* mice (**Fig. 3**) performing a Two-way ANOVA (factors: time and genotype) with post-hoc Multiple Comparison Procedure. The interaction p values of the ANOVA are the following: **Figure 3**: **A** and **B** p<<2×10^−16^, **C.** p = 5.3×10^−6^. The significant comparisons determined with the post-hoc Multiple Comparison Procedure were marked by asterisks in **Figure 3**. A likelihood ratio test for Negative Binomial generalized linear models [23] was used to compare the number of rows of PR nuclei in *sh1^+/−^* vs *sh1^−/−^* (**Fig. 4**). Statistical analyses were not required for data depicted in **Figure 5** **, S3, S4, S5** and **S6**. One-way ANOVA with post-hoc Multiple Comparison Procedure was used to make comparisons among groups in **Figure 6**. The p values of the ANOVA are the following: p = 4.6×10^−11^ (albino), p = 0.007 (pigmented). The significant comparisons determined with the post-hoc Multiple Comparison Procedure were marked by asterisks in **Figure 6**. A likelihood ratio test for Negative Binomial generalized linear models [23] was used to compare the number of correctly localized melanosomes (**Fig. 7** **)**. The significant comparisons determined with the post-hoc Multiple Comparison Procedure were marked by asterisks in **Figure 7**. The retinal recovery from light desensitization was compared in *hMYO7A*- vs *EGFP*-treated *sh1^−/−^* mice (**Fig. 8**) performing a Two-way ANOVA (factors: time and treatment) with post-hoc Multiple Comparison Procedure. The interaction p values of the ANOVA is p = 0.0003.The significant comparisons determined with the post-hoc Multiple Comparison Procedure were marked by asterisks and pound keys in **Figure 8**.

### Ethics Statement

This study was carried out in accordance with the Association for Research in Vision and Ophthalmology Statement for the Use of Animals in Ophthalmic and Vision Research and with the Italian Ministry of Health regulation for animal procedures. All procedures on mice were submitted to the Italian Ministry of Health; Department of Public Health, Animal Health, Nutrition and Food Safety on October 17th, 2011. The Ministry of Health approved the procedures by silence/consent, as per article 7 of the 116/92 Ministerial Decree. Surgery was performed under anesthesia and all efforts were made to minimize suffering. **Mice.** Mice were housed at the Institute of Genetics and Biophysics animal house (Naples, Italy) and maintained under 12-hour light/dark cycle (10–50 lux exposure during the light phase). Albino *shaker1^4626SB/4626SB^* mice (referred as *sh1^−/−^*) were imported from the Medical Research Council Institute of Hearing Research (Nottingham, UK) and maintained inbred; breedings were performed crossing heterozygous female with homozygous males. The albino *shaker1* mice used in this study were either homozygous *shaker1^4626SB/4626SB^* (*sh1^−/−^*) or heterozygous *shaker1^+/4626SB^* (*sh1^+/−^*). Pigmented *shaker1^4626SB/4626SB^* mice were imported from the Wellcome Trust Sanger Institute (Cambridge, UK) (a kind gift of Dr. Karen Steel) and back-crossed twice with CBA/Ca mice purchased from Harlan Italy SRL (Udine, Italy) to obtain heterozygous *sh1^+/−^* mice to expand the colony, then, they were maintained intercrossed; breedings were performed crossing heterozygous female with heterozygous males. The pigmented *sh1* mice used in this study were either homozygous *shaker1^4626SB/4626SB^ (sh1^−/−^*) or heterozygous *shaker1^+/4626SB^* (*sh1^+/−^*). All homozygous *sh1^−/−^* mice, whether albino or pigmented, showed at visual inspection hyperactivity, head-tossing, and circling behavior caused by vestibular dysfunctions. All mice were genotyped to confirm the visual inspection. The genotype for the *Myo7a^4626SB^* allele was performed by PCR analysis of genomic DNA (extracted from the mouse tip tail) followed by DNA sequencing. The primers used for the PCR amplification are the following: Fw1 (GTGGAGCTTGACATCTACTTGACC) and Rev3 (AGCTGACCCTCATGACTCTGC) which generate a product of 712 bp. Direct sequencing of the PCR product, performed using the Fw1 primer, confirmed that our lines of *sh1* mice carry the *Myo7a^4626SB^* allele which results in Gln720X [24]. In addition, since the CBA/Ca mice purchased from Harlan carry the *rd1-*PDEβ mutation we also genotyped the pigmented *sh1* mice used in this study for the *rd1*-PDEβ allele. PCR amplification on genomic DNA using the Rd1Fw (CACACCCCCGGCTGATCACTG) and Rd1Rev (CTGAAAGTTGAACATTTCATCAG) oligonucleotides gives a product of either 319 or 320 bp for the *wild type*- or *rd1-PDEβ* allele, respectively. Direct sequencing of the PCR products using the Rd1Rev primer as well as restriction enzyme digestion with DdeI allowed selecting pigmented *sh1* mice that did not carry homozygous *rd1-PDEβ* alleles. The RPE65 genotype was performed as previously described [9] and showed that both albino and pigmented *sh1* mice were homozygous for the *wild type* L450 allele.

### Electrophysiological Recordings

Before electrophysiological testing, mice were dark reared for three hours and anesthetized [25]. Flash electroretinograms (ERGs) were evoked by 10-ms light flashes generated through a Ganzfeld stimulator (CSO, Costruzione Strumenti Oftalmici, Florence, Italy) and registered as previously described [25]. ERGs and b-wave thresholds were assessed using the following protocol. Eyes were stimulated with light flashes increasing from −5.2 to +1.3 log cd*s/m^2^ (which correspond to 1×10^−5.2^ to 20.0 cd*s/m^2^) in scotopic conditions. The log unit interval between stimuli was 0.3 log from −5.4 to 0.0 log cd*s/m^2^_,_ and 0.6 log from 0.0 to +1.3 log cd*s/m^2^. For ERG analysis in scotopic conditions the responses evoked by 11 stimuli (from −4 to +1.3 log cd*s/m^2^) with an interval of 0.6 log unit were considered (**Fig. 1** **, S1A and S2**). To minimize the noise, three ERG responses were averaged at each 0.6 log unit stimulus from −4 to 0.0 log cd*s/m^2^ while one ERG response was considered for higher (0.0−+1.3 log cd*s/m^2^) stimuli. The time interval between stimuli was 10 seconds from −5.4 to 0.7 log cd*s/m^2^, 30 sec from 0.7 to +1 log cd*s/m^2^, or 120 seconds from +1 to +1.3 log cd*s/m^2^. a- and b-waves amplitudes recorded in scotopic conditions were plotted as a function of increasing light intensity (from −4 to +1.3 log cd*s/m^2^, **Fig. 1** **, S1 and S2**). The retinal sensitivity to light was assessed in scotopic conditions and measured as the lowest light stimulus able to evoke a b-wave-shaped response in triplicate (only reproducible responses were considered, **Fig. 2** **and S1B**), as previously described [26]. The photopic ERG was recorded after the scotopic session by stimulating the eye with ten 10 ms flashes of 20.0 cd*s/m^2^ over a constant background illumination of 50 cd/m^2^. To assess the recovery from light desensitization (**Fig. 3**) eyes were stimulated with 3 light flashes of 1 cd*s/m^2^ and then desensitized by exposure to constant light (300 cd/m^2^) for 3 minutes. Then, eyes were stimulated over time using the pre-desensitization flash (1 cd*s/m^2^) at 0, 5, 15, 30, 45 and 60 minutes post-desensitization. The recovery of rod activity was evaluated by performing the ratio between the b-wave generated post-desensitization (at the different time points) and that generated pre-desensitization. The recovery from light desensitization analysis after AAV injection (**Fig. 8**) was performed by a blinded operator (different from the one who has injected the animals) who did not know which eyes were injected with AAV-*hMYO7A* and which with control vectors. For the electrophysiological analysis the number of albino *sh1* mice analyzed was the following: Ganzfeld ERG study (**Fig. 1** **and S1**): 7 *sh1^+/−^* vs 8 *sh1^−/−^* at 3 months, 7 *sh1^+/−^* vs 8 *sh1^−/−^* at 6 months, 7 *sh1^+/−^* vs 11 *sh1^−/−^* at 12 months; light-sensitivity study (**Fig. 2** **and S1B**): 8 *sh1^+/−^* vs 8 *sh1^−/−^* at 3 and 6 months, 7 *sh1^+/−^* vs 11 *sh1^−/−^* at 12 months; recovery from light desensitization study (**Fig. 3**): 4 *sh1^+/−^* vs 4 *sh1^−/−^* at 2 months, 7 *sh1^+/−^* vs 8 *sh1^−/−^* at 6 months, and 9 *sh1^+/−^* vs 10 *sh1^−/−^* at 12 months. The number of pigmented *sh1* mice analyzed was the following: Ganzfeld ERG study (**Fig. S2**): 5 *sh1^+/−^* vs 4 *sh1^−/−^* between 6–7 months, 7 *sh1^+/−^* vs 4 *sh1^−/−^* at 12 months. The number of eyes varies in **Figures 1** **,** **2** **,** **3** **, S1A and S2A** based on whether one or both eyes of the same animal were analyzed or not due to either technical problems (i.e. corneal opacity and dryness) or recording problems.

### Generation of the pAAV2.1-*human MYO7A* Construct, AAV Vector Production and Purification

The pAAV2.1-CMV-*human MYO7A* (pAAV2.1-CMV-*hMYO7A*) construct was generated as previously described [27]. Briefly, the *human MYO7A* (*hMYO7A*) CDS (6648 bp) from isoform 1 (NCBI Reference Sequence NM_000260.3) was obtained by PCR amplification on commercially-available cDNA from human retina and cloned in the pAAV2.1-CMV-*EGFP* plasmid [28]. The pAAV2.1 plasmid contains the inverted terminal repeats (ITRs) from AAV2 which flank the expression cassette and that are required for AAV vector production [28]. The length of the AAV genome (including ITRs) encoded by the pAAV2.1-CMV-*hMYO7A* is 8107 bp. This oversize AAV *cis*-plasmid was used to produce both the AAV2/2 vectors for the *in vitro* experiments on HEK293 cells (**Fig. 5A**) as well as the AAV2/5 vectors for the *in vivo* experiments in *sh1^−/−^* mice (**Fig. 5B** **,** **6** **–** **8**). The pAAV2.1-CMV-*hMYO7A-HA* construct was generated by cloning the sequence of the human influenza hemagglutinin tag (HA) in frame with the *hMYO7A* CDS before the stop codon. The aminoacidic sequence of the HA-tag fused at the hMyo7a C-terminus is the following: MYDVPDYASL. The pAAV2.1-CMV-*hMYO7A-HA* plasmid was used to produce the AAV2/2 vectors used for the *in vitro* experiments on HEK293 cells depicted in **Figure S5**. The pAAV-CMV-*CEP290* ([27], genome length including ITRs: 8930 bp), pAAV2.1-CMV-iPS (sequence available upon request, genome length including ITRs: 7744 bp), and pAAV2.1-CMV-Kan (sequence available upon request, genome length including ITRs: 8734 bp) plasmids were used for the production of the oversize AAV2/5 vectors used in **Figure S4**. AAV vector preparations were produced by the TIGEM AAV Vector Core, by triple transfection of HEK293 cells followed by two rounds of CsCl2 purification [29]. For each viral preparation, physical titers [genome copies (GC)/mL] were determined by averaging the titer achieved by dot-blot analysis [30] and by PCR quantification using TaqMan [29] (Applied Biosystems, Carlsbad, CA, USA).

### AAV Vector Administration to *sh1* Mice

Adult (P28–P38) *sh1* mice were anesthetized with an intraperitoneal injection of 2 mL/100 g body weight of avertin [1.25% w/v of 2,2,2-tribromoethanol and 2.5% v/v of 2-methyl-2-butanol (Sigma-Aldrich, Milan, Italy)], then AAV vectors were delivered subretinally via a trans-scleral transchoroidal approach as described by Liang et al. [31]. All eyes were treated with 1 µL of vector solution supplemented with 40 µM calpain inhibitor (LnLL; Sigma-Aldrich, Milan, Italy) [27], [32], [33]. Albino *sh1^−/−^* mice (n = 5) analyzed in **Figures 5B** **and** **8** were injected in one eye (n = 4) with AAV2/5-CMV-*hMYO7A* (3×10^8^ GC) and in the contralateral eye (n = 4) with AAV2/5-CMV-*EGFP* (3×10^8^ GC) while one mice was injected with AAV2/5-CMV-*EGFP* (3×10^8^ GC) in both eyes. Eyes were analyzed by electrophysiology (**Fig. 8**) and then harvested for Western blot analysis at 15 months (**Fig. 5B**). Pigmented *sh1* mice were used for the analysis of melanosome localization (**Fig. 7A–B**) while both albino and pigmented *sh1* mice were used for the immunogold analysis (**Fig. 6A–B**). Pigmented *sh1^−/−^* mice were co-injected with AAV2/5-CMV-*hMYO7A* (4×10^8^ GC) and AAV2/5-CMV-*EGFP* (1×10^8^ GC) in one eye and with AAV2/5-CMV-*EGFP* (4×10^8^ GC) in the contralateral eye. Control pigmented *sh1^+/−^* mice were injected with AAV2/5-CMV-*EGFP* (4×10^8^ GC) in both eyes (**Fig. 6A–B** **and** **7A–B**). Albino *sh1^−/−^* mice were co-injected with AAV2/5-CMV-*hMYO7A* (3×10^8^ GC) and AAV2/1-CMV-*human Tyrosinase* (3×10^7^ GC) [26] in one eye, and AAV2/5-CMV-*EGFP* (3×10^8^ GC) plus AAV2/1-CMV-*hTyrosinase* (3×10^7^ GC) in the contralateral eye (**Fig. 6A**). Control albino *sh1^+/−^* mice were co-injected with AAV2/5-CMV-*EGFP* (3×10^8^ GC) and AAV2/1-CMV-*human Tyrosinase* (3×10^7^ GC) in both eyes (**Fig. 6A**). AAV2/5-CMV-*EGFP* or AAV2/1-CMV-*human Tyrosinase* [26] were co-injected in pigmented and albino *sh1* eyes, respectively, to mark the RPE in the injected area in order to dissect and use the transduced part of the eyecup to perform the rescue analyses (**Fig. 6A–B** **and** **7A–B**). The left and right eyes were randomly assigned to either the group treated with AAV-*hMYO7A* or to the group treated with the control AAV-*EGFP* vector. In addition, affected *sh1^−/−^* mice were randomly allocated (i.e. without following the sequential animal numbering) to the different treatment groups.

### Transfection and AAV Infection of HEK293 Cells

HEK293 cells (American Type Culture Collection, Manassas, VA, USA) were maintained in Dulbecco’s modified Eagle’s medium (DMEM, GIBCO, Invitrogen S.R.L., Milan, Italy) containing 10% fetal bovine serum (FBS, GIBCO, Invitrogen S.R.L., Milan, Italy). For the experiment depicted in **Figures 5A and S** **5**, cells were plated in 6-well plates (2×10^6^ cells/well), 24 hours later cells were transfected with 1 µg of ΔF6 helper plasmid by calcium phosphate method, after 5 hours cells were carefully washed with serum-free DMEM and incubated in serum-free DMEM for 2 hours with 1×10^5^ GC/cell of either AAV2/2-CMV-*EGFP*, AAV2/2-CMV-*hMYO7A* or AAV2/2-CMV-*hMYO7A-HA*; then, 2 mL of DMEM-10%FBS were added to the cells. Thirty-six hours later, cells were washed twice with phosphate buffered saline and harvested by scraping, lysed and used for Western blot analysis. To generate a positive control, cells (from one well of a 6-well) were transfected with 1.3 µg of either pAAV2.1-CMV-*hMYO7A* or pAAV2.1-CMV-*hMYO7A-HA* plasmid by calcium phosphate method, washed after 5 hours and then harvested 36 hours later by scraping.

### Southern Blot Analyses of AAV Vector DNA

Alkaline Southern blot was used to analyze the size of the single-stranded DNA packaged in AAV capsids. Three x10^10^ GC of viral DNA were extracted from AAV particles. To digest unpackaged genomes, the vector solution was resuspended in 240 µL of PBS pH 7.4 1X (GIBCO, Invitrogen S.R.L., Milan, Italy) and then incubated with 1 U/µL of DNase I (Roche, Milan, Italy) in a total volume of 300 µL containing 40 mM TRIS-HCl, 10 mM NaCl, 6 mM MgCl2, 1 mM CaCl2 pH 7.9 for 2 hours at 37°C. The DNase I was then inactivated with 50 mM EDTA, followed by incubation with proteinase K and 2.5% *N*-lauroyl-sarcosil solution at 50°C for 45 minutes to lyse the capsids. The DNA was extracted twice with phenol-chloroform and precipitated with 2 volumes of ethanol 100% and 10% sodium acetate (3 M, pH 7). Alkaline agarose gel electrophoresis and blotting were performed as previously described [34]. Ten µL of the 1 kb DNA ladder (N3232L, New England Biolabs, Ipswich, MA, USA) were loaded as molecular weight marker. A 768 bp double strand DNA fragment (corresponding to the CMV promoter from pAAV2.1-CMV-*EGFP* plasmid) was radio-labeled with [α-32]-CTP using the Amersham Rediprime II DNA labeling System (GE Healthcare Europe, GmbH, Milan, Italy) and used as probe. Prehybridization and hybridization were performed at 65°C in Church buffer [34] for 1 hour and overnight, respectively. Then, the membrane (Whatman Nytran N, charged nylon membrane, Sigma-Aldrich, Milan, Italy) was washed for 30 minutes in SSC 2X-0.1% SDS and for 30 minutes in SSC 0.5X-0.1% SDS at 65°C, and then for 30 minutes in SSC 0.1X-0.1% SDS at 37°C. The membrane was then analyzed by X-ray autoradiography using Amersham Hyperfilm™ MP (GE Healthcare Europe, GmbH, Milan, Italy).

### Analysis of Myo7a Expression by Western Blot

HEK293 cells infected with AAV (**Fig. 5A and S** **5**), eyecups from albino *sh1^−/−^* mice injected with AAVs (**Fig. 5B**) and eyecups from albino *sh1^−/−^* and *sh1^+/−^* mice (**Fig. S6**) were lysed in RIPA buffer (50 mM Tris-Hcl pH 8.0, 150 mM NaCl, 1% NP40, 0.5% Na-Deoxycholate, 1 mM EDTA pH 8.0, 0.1% SDS), supplemented with protease inhibitors (Complete Protease inhibitor cocktail tablets, Roche, Milan, Italy) and 1 mM phenylmethylsulfonyl fluoride. Cell and eyecup lysates were separated by 7% SDS-polyacrylamide gel electrophoresis and immunoblotted using either a polyclonal anti-Myo7a antibody (Primm Srl, Milan, Italy; working dilution 1∶500) generated using a peptide corresponding to aminoacids 941–1070 of the human Myo7a protein (**Fig. 5**) or a polyclonal anti-HA antibody (PRB-101P-200, HA.11, Covance, working dilution 1∶2000). We confirmed by Western blot analysis that the anti-Myo7a antibody efficiently recognizes both the murine Myo7a protein in eyecup lysates from *sh1^+/−^* mice (**Fig. S6**) as well as the human Myo7a protein from cells transfected with the pAAV2.1-CMV-*hMYO7A* plasmid (**Fig. 5A**). The anti-Filamin A (catalog#4762, Cell Signaling Technology, Boston, MA) and the anti-Dysferlin (Dysferlin, clone Ham1/7B6, MONX10795, Tebu-bio, Le-Perray-en-Yvelines, France) antibodies were used to normalize the SDS-PAGE loading of cell and eyecup lysates, respectively.

### Histology and Light Microscopy

To count the rows of photoreceptor (PR) nuclei in the outer nuclear layer (ONL, **Fig. 4**) eyes from *sh1* mice were enucleated, fixed in 4% paraformaldehyde over-night and infiltrated with 30% sucrose over-night. Then, the cornea and the lens were dissected and the eyecups were embedded in optimal cutting temperature compound (O.C.T. matrix, Kaltek, Padua, Italy). Serial eyecup cryo-sections (10 µm thick) were cut along the horizontal meridian and progressively distributed on slides so that each slide contained representative sections from the whole eye. Sections were stained with hematoxylin and eosin (Richard-Allen Scientific, Kalamazoo, MI, USA) according to standard procedures. The rows of PR nuclei were counted by a blinded observer who did not know which retinas were from *sh1^+/−^* and which were from *sh1^−/−^* mice. More specifically, the eyes were collected and the slides were numbered by a person different from the observer who made the measurements. The observer analyzed 3 sections/eye by light microscopy at 40X magnification. Measurements were taken at 500, 1000, and 1500 µm from the optic nerve head both temporally and nasally. The number of *sh1* mice analyzed in **Figure 4** is the following: 3 *sh1^+/−^* vs 3 *sh1^−/−^* (albino, 6 months); 5 *sh1^+/−^* vs 6 *sh1^−/−^* (albino, 12 months); 4 *sh1^+/−^* vs 3 *sh1^−/−^* (pigmented, 6–7 months); 10 *sh1^+/−^* vs 3 *sh1^−/−^* (pigmented, 12 months). To analyze the melanosome localization in the RPE of pigmented *sh1^−/−^* or *sh1^+/−^* mice (**Fig. 7A–B**), eyes were enucleated 4 months following the AAV subretinal injection, fixed in 2% glutaraldehyde-2% paraformaldehyde in 0.1 M phosphate buffer over-night, rinsed in 0.1 M phosphate buffer and dissected under a florescence microscope. The EGFP-positive portions of the eyecups were embedded in Araldite 502/EMbed 812 (catalog #13940, Araldite 502/EMbed 812 KIT, Electron Microscopy Sciences, Hatfield, PA, USA). Semi-thin (0.5-µm) sections were transversally cut on a Leica Ultratome RM2235 (Leica Microsystems, Bannockburn, IL, USA), mounted on slides and stained with Epoxy tissue stain (catalog #14950, Electron Microscopy Sciences, Hatfield, PA, USA). Melanosomes were counted by a blinded observer who did not know which retinas were treated with AAV-*hMYO7A* and which with control vectors. More specifically, the eyes were collected and the slides were numbered by a person different from the observer who counted the melanosomes. The observer analyzed 10 different fields/eye under a light microscope at 100X magnification. Retinal pictures were captured using a Zeiss Axiocam (Carl Zeiss, Oberkochen, Germany). The number of *sh1* mice analyzed in **Figure 7A** is the following: 3 *sh1^+/−^* vs 4 *sh1^−/−^*.

### Immunogold and Electron Microscopy

The data showed in **Figure 6A** were obtained from albino and pigmented *sh1^−/−^* and *sh1^+/−^* mice. To quantify Rhodopsin localization to the connecting cilium of *sh1* photoreceptors, eyes were enucleated from albino *sh1* mice at 4 months following AAV injection and from pigmented *sh1* mice at 4 or 7 months following AAV injection. Eyes were fixed in 0.2% glutaraldehyde-2% paraformaldehyde in 0.1 M PHEM buffer pH 6.9 (240 mM PIPES, 100 mM HEPES, 8 mM MgCl_2_, 40 mM EGTA) for 2 hours and then rinsed in 0.1 M PHEM buffer. Then, eyes were dissected under a fluorescence or light microscope to select the EGFP- or tyrosinase-positive portions of the eyecups, respectively, which were subsequently embedded in 12% gelatin. Cryo-sections (50 nm) were cut using a Leica Ultramicrotome EM FC7 (Leica Microsystems, Bannockburn, IL, USA) and extreme care was taken to align photoreceptor connecting cilia longitudinally. The anti-Rhodopsin antibody (1∶100 1D4, ab5417, Abcam, Cambridge, UK) was used for immunogold labeling. The quantification of gold density of Rhodopsin in the connecting cilia was performed by a blinded observer who did not know which retinas were treated with AAV-*hMYO7A* and which with control vectors. More specifically, the eyes were collected and the slides were numbered by a person different from the observer who quantified gold density. The analysis was performed using the iTEM software (Olympus SYS, Hamburg, Germany) according to a previously described method [35]. Briefly, a morphometric grid with a 100 nm mesh was placed over images of photoreceptors. Touch counts module of the iTEM software was used to quantify: i. the number of gold particles in the cilium and ii. the number of intersections between grid and cilium membrane. Gold density was expressed in arbitrary units (gold particles per intersection). The number of *sh1* mice analyzed in **Figure 6A** is the following: 2 *sh1^+/−^* vs 2 *sh1^−/−^* (albino); 2 *sh1^+/−^* vs 2 *sh1^−/−^* (pigmented).

### Optical Coherence Tomography (OCT) and and Scanning Laser Ophthalmoscopy (SLO)

Mice were anaesthetized by intraperitoneal injection of a combination of ketamine (Virbac, Carros, France) at 150 mg/kg body weight and xylazine (12.5 mg/kg; KVP Pharma und Veterinaer-Produkte GmbH) at 10 mg/kg body weight. Pupils were dilated with tropicamide eye drops (Mydriaticum Stulln, Pharma Stulln, Stulln, Germany). In vivo imaging was performed as previously described [36]. Briefly, SLO and SD-OCT imaging were performed with a Spectralis™ HRA+OCT (Heidelberg Engineering GmbH, Heidelberg, Germany). This device features a superluminescent diode at 870 nm as low coherent light source. Each A-scan was taken at a speed of 40.000 scans per second and each B-scan contains up to 1536 A-scans. The images were taken with the equipment set of 30° field of view and with the software Heidelberg Eye Explorer (HEYEX version 3.2.1.0, Heidelberg, Germany) and they were processed with Corel Draw X3 (Corel Corporation, Ottawa, ON Canada). Age-matched C57BL/6 mice (purchased from Charles River Laboratories International, Inc.,Wilmington, MA, USA) were kept under a 12 h∶12 h light-dark cycle (60 lux) and had food and water *ad libitum*. All procedures were performed with permission of the local authorities (Regierungspraesidium, Tübingen, Germany).

## Results

### B-wave Electroretinogram is Decreased in Albino *sh1^−/−^* Mice

In all our studies we compared *sh1^−/−^* mice, whether albino or pigmented, to their heterozygous (*sh1^+/−^*) littermates. Ganzfeld flash electroretinograms (ERGs) in both photopic and scotopic conditions were recorded in albino *sh1^−/−^* and *sh1^+/−^* mice at 3, 6 and 12 months of age (**Fig. 1** **and S1**). At 3 months, no major abnormalities in the a- and b-wave amplitudes were observed in *sh1^−/−^* compared to age-matched *sh1^+/−^* mice (**Fig. S1A**). However a significant scotopic b-wave reduction was observed in affected *sh1^−/−^* mice compared to heterozygous controls at 6 months (20% reduction at 1.3 log) and 12 months (27% reduction at 1.3 log, **Fig. S1A and** **Table 1**). No a-wave abnormalities were instead observed in albino affected mice up to 12 months compared to heterozygous littermates (**Fig. 1A**). Notably, in both affected and control albino *sh1* mice a- and b-waves declined over time (**Table 1**). From 6 to 12 months of age the maximum b-wave amplitude (evoked with 20 cd*s/m^2^ in scotopic conditions) decreased of 40% in *sh1^+/−^* (ANOVA p value<<2×10^−16^) and of 45% in *sh1^−/−^* (ANOVA p value = 1.6×10^−9^) (**Table 1**) while the maximum a-wave amplitude decreased of 23% in *sh1^+/−^* (p value = 0.03) and 28% in *sh1^−/−^* (p value = 0.0075) (**Table 1**). In pigmented *sh1^−/−^* and *sh1^+/−^* mice, we recorded ERGs between 6 and 7 months and at 12 months of age and did not observe significant differences in affected mice compared to controls (**Fig. S2 and** **Table 1**). Differently from albino *sh1* mice, both pigmented *sh1^+/−^* and *sh1^−/−^* mice did not show significant maximum ERG reduction from 6–7 to 12 months of age (**Table 1**). Interestingly, the b-wave defects identified by the ERG analysis in albino *sh1^−/−^* showed that the albino but not the pigmented background negatively influenced retinal function of *Myo7a* deficient mice (**Fig. 1A, S** **2 and** **Table 1**) as observed in previous studies performed in other pigmented *shaker1* lines [7], [9].

**Table 1:** Maximum a- and b-wave amplitudes and rows of photoreceptor nuclei in the outer nuclear layer of *sh1* mice.

	MAX a-wave (µV)^a^				MAX b-wave (µV)^a^				PR rows in the ONL (n)			
	Albino		Pigmented		Albino		Pigmented		Albino		Pigmented	
	*sh1* ^+/−^	*sh1* ^−/−^	*sh1* ^+/−^	*sh1* ^−/−^	*sh1* ^+/−^	*sh1* ^−/−^	*sh1* ^+/−^	*sh1* ^−/−^	*sh1* ^+/−^	*sh1* ^−/−^	*sh1* ^+/−^	*sh1* ^−/−^
3 M	284±16(14)^b^	348±10(16)	nd	nd	838±29(14)	834±26(16)	nd	nd	nd	nd	nd	nd
6 M	289±21(13)	297±16(15)	403±63(8)	329±34 (8)	756±17(13)	610±25(15)	665±68(8)	529±30(8)	8.5±0.1(3)	8.8±0.1(3)	9±0.2 (4)	9±0.2(3)
12 M	222±21(14)	213±22(22)	355±25(11)	307±44 (7)	457±35(14)	337±22(22)	589±38(12)	513±73(7)	8.2±0.1(5)	8.3±0.1(6)	8.8±0.2(10)	8.9±0.3(3)

^a^Maximum a- and b-wave amplitudes are those recorded at 20.0 cd*s/m^2^ (+1.3 log units) in scotopic conditions, as shown in **Figures 1**, **S1** and **S2**.

b The number of eyes analyzed is showed in brackets. nd: not determined; ONL: outer nuclear layer; n: mean number of rows of photoreceptor (PR) nuclei in the ONL; data are showed as mean±SEM.

### Light Sensitivity Decreases with Age in Albino *sh1^−/−^* Mice

Based on the ERG reduction measured in albino *sh1^−/−^* mice we further investigated their retinal function. Rod sensitivity to light was evaluated in scotopic conditions in dark-adapted albino *sh1^−/−^* and *sh1^+/−^* mice by measuring the lowest light stimulus able to evoke a b-wave-shaped response. Rod sensitivity is low when the intensity of the light stimulus (defined as b-wave threshold) that evokes the b-wave is high. Notably, the b-wave thresholds were significantly higher in albino *sh1^−/−^* mice compared to the corresponding *sh1^+/−^* controls (**Fig. 2**). In particular albino *sh1^−/−^* showed an early onset reduction of rod sensitivity to light (**Fig. 2** **and S1B**) that further declined with age reaching about 67% of heterozygous controls at 12 months. Therefore, albino *sh1^−/−^* mice presented with a decrease in rod sensitivity to light.

### Recovery from Light Desensitization is Decreased in Albino *sh1^−/−^* Mice

We then evaluated the ability of rod photoreceptors to recover from light desensitization in albino *sh1^−/−^* mice and corresponding controls at 2 (**Fig. 3A**), 6 (**Fig. 3B**) and 12 (**Fig. 3C**) months of age. We measured the b-wave amplitude evoked by a light stimulus of 1 cd*s/m^2^ before and after rod desensitization. To desensitize rods we exposed the eyes to pre-adapting light for 3 minutes, after desensitization we stimulated rods with a light stimulus of 1 cd*s/m^2^ over time (0, 5, 15, 30 45 and 60 minutes) and measured the b-wave amplitude evoked (**Fig. 3**). Sixty minutes after desensitization albino *sh1^+/−^* mice generated a b-wave similar to the one generated before desensitization, therefore the ratio between the post- and pre-bleaching b-waves is around 1 (**Fig. 3**). The ability of the albino *sh1* ^+/−^ retina to recover from light desensitization decreased with age (**Fig. 3**): b-wave ratio is 0.97±0.01 (mean±SEM) at 2 months and 0.82±0.04 (mean±SEM) at 12 months (ANOVA p value = 0.03). Remarkably, albino *sh1* ^−/−^ mice showed an early severe impairment of the rod recovery from light desensitization (**Fig. 3**), indeed at 2 months of age the affected mice only recovered about 50% of the pre-desensitization b-wave at 60 minutes (**Fig. 3**). Notably, the impairment in recovery from light desensitization of the *sh1* ^−/−^ retina is stable from 2 to 12 months of age (mean b-wave ratio±SEM = 0.50±0.03 at 2 months vs 0.54±0.02 at 12 months, ANOVA p value = 0.3) (**Fig. 3**). This early and marked impairment of recovery from light desensitization by the *sh1^−/−^* retina is the first important functional defect associated with *Myo7a* deficiency.

### No Significant PR Degeneration in Albino and Pigmented *sh1^−/−^* Mice

To date no PR degeneration has been observed in *shaker1* murine lines that bear various loss-of-function mutations in the *Myo7a* gene, including the *sh1* ^−/−^ mice we used in our studies [8], [9], [10]. To confirm this in our lines of albino and pigmented *sh1* ^−/−^ mice, we counted the rows of PR nuclei in the outer nuclear layer (ONL) using retinal sections from affected and control *sh1* mice that were previously subjected to electrophysiological analyses (**Fig. 4** **and** **Table 1**). The rows of PR nuclei of both albino and pigmented *sh1^−/−^* mice were not significantly different from those of control *sh1^+/−^* mice at 6–7 and 12 months of age (**Fig. 4** **and** **Table 1**). In addition, retinal optical coherence tomography (OCT) and scanning laser ophthalmoscopy (SLO) imaging (**Fig. S3**) were performed in a subset of 12 month-old pigmented *sh1* mice before they were sacrificed for the histological analysis shown in **Figure 4**. The OCT and SLO analyses showed that the ONL thickness as well as the retinal architecture was not different between *sh1^−/−^* and *sh1^+/−^* mice (**Fig. S3**), thus confirming the data from the histological analysis (**Fig. 4**). Therefore the electrophysiological defects we observed in the albino *sh1^−/−^* retina are not due to significant PR cell loss but are potentially due to functional impairments that result from lack of Myosin7a-mediated motor kinesis.

### AAV-mediated Delivery of Human *MYO7A* Ameliorates the Morphological and Functional Defects of the *sh1^−/−^* Retina

To rescue the retinal phenotype of adult *sh1^−/−^* mice we performed *MYO7A* gene replacement using adeno-associated viral vectors (AAVs), which efficiently transduce both RPE and PRs [37], [38]. We generated AAV2/2 and 2/5 vectors by using the pAAV2.1-CMV-*hMYO7A cis*-plasmid that contains the complete human *MYO7A* (*hMYO7A*) coding sequence (6648 bp) under the control of the ubiquitous cytomegalovirus (CMV) promoter, which is active in both PRs and RPE [37], [38]. This 8.1 Kb expression cassette exceeds the canonical AAV cargo capacity and results in the generation of the so called “oversize” AAV vectors [27], [32], [33], [39], [40], [41], [42], [43], [44], [45]. Oversize AAV vectors have been reported to contain genomes of heterogeneous size [41], [42], [43], [44], [45], [46], [47] which may preclude their clinical application. Indeed, we have analyzed by alkaline Southern Blot the size of DNAse I-resistant viral genomes from oversize AAV2/5 vectors generated by using various pAAV2.1 *cis*-plasmids containing large transgenes (ranging from 7.7 to 8.9 Kb in size, see Materials and Method section). While we observe a low amount of encapsidated genomes >5 kb, which appear more heterogeneous in size than what we have observed in the past [27], we have found a large population of smaller genomes ≤5 kb, similarly to what reported by others (**Fig. S4**) [41], [42], [43], [44], [45], [46]. Similar results were obtained analyzing both AAV2/2- and 2/5-CMV-*hMYO7A* vectors (data not shown). Despite this limitation, oversize AAV vectors are useful research tools for large gene expression in photoreceptors and retinal pigment epithelium *in vivo* [27], [48].

To assess the expression of hMyo7a *in vitro* we used AAV2/2 vectors which infect HEK293 cells more efficiently than AAV2/5 [49]. Oversize AAV2/2 vectors were used to infect HEK293 cells followed by Western blot analyses on cell lysates using anti-Myo7a antibody (**Fig. 5A**). As negative control we infected cells with AAV2/2 vectors that encode for the enhanced green fluorescent protein (EGFP), while as positive control we transfected HEK293 cells with the same pAAV2.1-CMV-*hMYO7A cis*-plasmid used for oversize AAV production (**Fig. 5A**). Notably, infection of HEK293 cells with AAV2/2-CMV-*hMYO7A* resulted in robust expression of full-length hMyo7a protein (**Fig. 5A**). We obtained a similar result by Western blot analysis with anti-HA antibodies of cells infected with AAV2/2-CMV-*hMYO7A-HA* vector which contains the HA tag sequence at the 3′end of the *hMYO7A* coding sequence. This allows us to exclude that the strong AAV-mediated expression of hMyo7a *in vitro* may be due to the integration of the transgene *hMYO7A 5′* end containing the CMV promoter at the endogenous HEK293 *MYO7A* locus (**Fig. S5**). Then, we injected subretinally AAV2/5-CMV-*hMYO7A* in one eye and AAV2/5-CMV-*EGFP* in the contralateral eye of albino *sh1^−/−^* mice (**Fig. 5B**). Fifteen months after injection, the eyecups were harvested, lysed and analyzed by Western blot analysis using the anti-Myo7a antibody. We observed by Western blot analysis that the full-length hMyo7a protein was expressed at various levels in the eyecups of *sh1^−/−^* mice injected with AAV2/5-CMV-*hMYO7A*, but not with AAV2/5-CMV-*EGFP* (**Fig. 5B**). This hMyo7a expression presumably derives from transduction of both RPE and photoreceptors since we have previously reported that oversize AAV2/5 vectors injected subretinally transduce both cell types [27].

We then evaluated the therapeutic efficacy of AAV-mediated *hMYO7A* transfer to the *sh1^−/−^* retina (**Fig. 6** **,** **7** **and** **8**). We initially analyzed the subcellular localization of Rhodopsin (rho) in *sh1^−/−^* PRs by immunogold analysis (**Fig. 6A–B**). Consistently with what previously reported [14], we found a significant accumulation of rho at the connecting cilium of *sh1^−/−^* mice compared to their heterozygous littermates (**Fig. 6A–B**). Following AAV2/5-*hMYO7A* delivery the number of rho molecules at the connecting cilium of PRs in the transduced area was significantly lower than in *sh1^−/−^* mice injected with AAV2/5-*EGFP* (**Fig. 6A–B**). Then, we evaluated the localization of melanosomes in the RPE of pigmented *sh1^−/−^* and *sh1^+/−^* mice treated with AAVs by histological analysis of semi-thin retinal sections (**Fig. 7A–B**). Melanosome counts (**Fig. 7B**) and representative retinal images (**Fig. 7B**) show that differently from *sh1^+/−^*, the melanosomes of *sh1^−/−^* mice do not enter in the RPE microvilli, and that this is corrected by AAV2/5-CMV-*hMYO7A* injection (**Fig. 7A–B**). **Figure 7B**, shows that in the area treated with AAV2/5-CMV-*hMYO7A* many RPE cells present correctly localized melanosomes (**Fig. 7B**). Although the number of AAV-*hMYO7A*-treated retinas is low, the apical position of melanosomes in the sections from this retina appears to be due to *hMYO7A* gene replacement as none of the *EGFP*-treated eyes shows a single correctly-localized melanosome (**Fig. 7A–B**). Finally, we measured the ability of the albino *sh1^−/−^* retina to recover from light desensitization following injection of either AAV2/5-CMV-*hMYO7A* or AAV2/5-CMV-*EGFP* as control (**Fig. 8**). This analysis can not be performed in pigmented *sh1* mice which do not have defects in the recovery from light desensitization compared to *sh1^+/−^* controls (data not shown). At 6 months post-injection (**Fig. 8**) retinal recovery from light desensitization of *sh1^−/−^ hMYO7A*-treated eyes was significantly different from that of *EGFP*-treated eyes (ANOVA p value = 9.7×10^−9^) but not from those of *sh1^+/−^* eyes (ANOVA p value = 0.18; **Fig. 8**). A similar result was observed one month after AAV2/5-CMV-*hMYO7A* subretinal administration in 7-month-old *sh1^−/−^* mice (data not shown). Thus, recovery from light desensitization is a consistent and sensitive test to assess the outcome on retinal function of experimental therapies for USH1B in *sh1^−/−^* mice.

## Discussion

*Shaker1* mice are the best characterized model of USH1B, one of the most common severe forms of syndromic retinitis pigmentosa. While *sh1^−/−^* mice have clear ultrastructural PR and RPE anomalies, no important functional defects of the *sh1^−/−^* retina have been identified yet [7], [8], [9], [14], [16]. Here we show that *Myo7a* deficiency causes severe retinal dysfunctions in albino *sh1^−/−^* mice.

The most significant retinal functional anomaly we found in albino *sh1^−/−^* mice is the 50% reduction in recovery from light desensitization that is evident from 2 months of age. Light desensitization saturates active Rhodopsin molecules in the PR OSs, therefore the recovery of rod dark adaptation mainly relies on the efficient regeneration of Rhodopsin that depends on: i. transport of new opsin molecules from the PR connecting cilium to their OS; and ii. recycling of 11-*cis*-retinal (the opsin cofactor) by the visual cycle [50]. It is likely that the slower opsin transport as well as the slower turnover of PR OS reported in *sh1^−/−^* mice [14] contributes to delayed recovery after light desensitization. RPE defects could additionally contribute to the impairment in the recovery from light desensitization, since Lopes et al. have recently shown that the expression of Myo7a in RPE cells is required for the proper activity of the RPE65 isomerase which is a key enzyme for the generation of the 11-*cis*-retinal in the visual cycle [9]. Thus, both PR and RPE defects of *sh1^−/−^* mice could have a functional counterpart in the inability of the *sh1^−/−^* retina to recover from light desensitization. Independently of the mechanism, reduced levels of Myo7a negatively influence the ability of rods to recover from light desensitization in albino *sh1* mice. Whether this is common to other *shaker1* lines, that bear alleles other than the 4626SB, remains to be tested. In addition, it remains to be assessed how this defect extrapolates to the human USH1B retina that, differently from the mouse, presents with severe retinitis pigmentosa characterized by photoreceptor degeneration and decreased Ganzfeld ERGs [1], [20]. No abnormalities in the dark adaptation kinetics have been recently found in USH1B patients [20]. However, delayed recovery of rod sensitivity after light desensitization has been reported in carrier and affected USH patients [51], [52] as well as in individuals with retinitis pigmentosa [53], [54], [55]. In any case the recovery from light desensitization represents an important functional read-out to test the efficacy of experimental therapies for the USH1B retina at the pre-clinical level. We also found that rod light sensitivity is significantly reduced in affected *sh1* mice compared to heterozygous controls. As Rhodopsin transport to the outer segment has been reported to be reduced in the absence of Myo7a [14], it is possible that more photons are required to evoke a b-wave response in presence of a lower number of Rhodopsin molecules. A previous study in pigmented *sh1^−/−^* mice on a mixed background reported no significant reduction of light sensitivity but attenuated a- and b-wave amplitudes at higher light intensities (about 20% reduction at 0.0 log) [7]. We did observe a subtle a- and b-wave reduction at 0.0 log in pigmented *sh1^−/−^* compared to *sh1^+/−^* but this was not statistically significant. We think that this can be ascribed to the difference in the *sh1^−/−^* background and/or to the very subtle entity of the ERG reduction in pigmented *sh1^−/−^* mice, which can be appreciated only when a very high number of eyes are analyzed.

On the other hand we observed a mild (approximately 20%), although significant, Ganzfeld ERG b-wave reduction in albino *sh1^−/−^* mice after 6 months of age. However, we consider the entity of the ERG reduction observed in albino *sh1^−/−^* mice too small to be reliably used to assess rescue of retinal function in gene therapy studies. Notably, the ERG phenotype of *sh1^−/−^* mice is not due to progressive photoreceptor degeneration since we did not found significant photoreceptor cell loss in affected mice compared to controls. The molecular basis of the ERG reduction in *sh1^−/−^* mice has not been thoroughly dissected yet however, it is likely that the *sh1^−/−^* PR and RPE defects, as well as the impaired rod dark adaptation could account for the Ganzfeld ERG phenotype. Recently Peng et al. [10] reported that the translocation of transducin from the OS to the inner segment of PRs is delayed in *Myo7a^sh1-11J^* mice. If this holds true for the albino *sh1^−/−^* mice we used in our studies, it could contribute to retinal dysfunction since transducin is a key component of the phototransduction cascade and its translocation is important for PR adaptation to various light conditions [50], [56]. As alternative explanation for the ERG reduction, we excluded the difference in size between *sh1^−/−^* and *sh1^+/−^* as this difference in size is present also on the pigmented background which does not present significant ERG reduction.

Interestingly, as previously reported in other studies [8], [9] we observed that *Myo7a* deficiency did not impact on PR survival since the rows of PRs nuclei of affected mice were not significantly different from those of heterozygous controls from both albino and pigmented lines. Notably, we observed that the albino background which is known to exacerbate PR degeneration in other murine models of PR disease [57] did not influence PR cell loss in *sh1^−/−^* mice. This may be explained by the lower activity of RPE65 found in *sh1^−/−^* mice [9] which may render *Myo7a-*deficient PRs more resistant to the damaging effect of ambient light as it makes them extremely resistant to acute light damage [9].

The *sh1^−/−^* mice are crucial to evaluate the efficiency and safety of experimental therapies for USH1B. Retinitis pigmentosa in USH1B is severe leading to blindness in the first decades of life [1]. Gene therapy with vectors derived from AAV is being evaluated in patients with an inherited form of childhood blindness and the safety and the efficacy results from three independent clinical trials are extremely encouraging [58].

However, AAV vectors have a cargo capacity considered to be limited to 4.7 Kb [41], [42], [43], [44], [45], [46], [47] and this is a major limitation when delivering large genes like *MYO7A*. This can be overcome by either generating oversize AAV vectors by using a plasmid containing a large gene expression cassette [27], [32], [33], [39], [40], [41], [42], [43], [44], [45] or by splitting a large gene into two halves each contained in a separate, regular size AAV vector (dual-AAV vectors [59]). The potential of dual-AAV vectors in the retina has been underlined using a reporter gene [60]. However no therapeutic applications of this platform in the retina have been reported yet. Gene transfer with oversize AAV vectors has resulted in expression of large proteins and subretinal administration of oversize AAV2/5 vectors has allowed expression in both RPE and photoreceptors of the large *MYO7A* and *ABCA4* [27], [48] genes at therapeutic levels in mice.

In a previous study [27] we have shown that oversize AAVs express the full-length hMyo7a protein in RPE cultures from *sh1^−/−^* mice. In the present study we report that AAV-mediated expression of hMyo7a in the retina of adult *sh1^−/−^* mice rescues PR Rhodopsin transport and RPE melanosome localization and improves the recovery profile from light desensitization, thus validating this as a sensitive electrophysiological test to assess improvement of retinal function in pre-clinical gene therapy studies for the USH1B retina.

Our current data as well as those published by others [41], show that the size of the genome contained within oversize AAV particles is highly heterogeneous, and mostly smaller than expected. However, after infection of target cells, AAV DNA is found to have an homogenous size which corresponds to the large transgene expression cassette [27], [45]. Accordingly, the corresponding mRNA and protein are full size without evidence so far of truncated products [44], [45]. Therefore, based on the results presented here and by others the molecular mechanism underlying oversize AAV-mediated transduction remains elusive. Large gene transduction may derive from: i. full-length genomes contained in the capsid [27], [32], [39], [40]; ii. re-assembly in transduced cells of truncated genomes [41], [43], [44], [45], [46], [47] produced during oversize genome encapsidation; iii. a combination of the two previous mechanisms. Independently of the mechanism underlying oversize AAV vector transduction, the heterogeneous size of their genomes may limit their therapeutic applications. However oversize AAVs represents valuable research tools for gene expression in the retina and more specifically in adult photoreceptors where other vector systems may have limited access [61]. Indeed previous gene transfer studies with lentiviral vectors which have higher cargo capacity than AAV but poor PR transduction ability [62], [63] have shown that *hMYO7A* gene delivery to the newborn *sh1^−/−^* retina results in therapeutic efficacy [2]. However, the ability of lentiviral vectors to efficiently transduce adult murine PRs remains to be demonstrated. In conclusion, the current study provides important tools to assess the rescue of retinal function in *sh1^−/−^* mice in pre-clinical gene therapy studies and further validates *sh1^−/−^* as a model of USH1B. In addition, it provides proof-of-principle that gene transfer improves the phenotype of the *sh1^−/−^* retina including its functional abnormality. Once a vector platform capable of safely and efficiently transfer large genes to photoreceptors and RPE will be made available, our data prompt its testing in the *sh1^−/−^* retina for further development of gene therapy for USH1B.

## Supporting Information

Figure S1 **Ganzfeld electroretinograms (A) and retinal light sensitivity (B) of albino** ***sh1*** **mice at 3 months of age. A–B**. Data are presented as mean±SEM, n indicates the number of eyes analyzed, the arrows point at the photopic ERG. *p value<0.05. More details on the statistical analysis including specific statistical values can be found in the Statistical analysis paragraph of the Materials and Methods section.
(TIF)

Figure S2 **Ganzfeld electroretinograms in pigmented** ***sh1*** **mice. A**. Mean a- (top panels) and b-waves (bottom panels) from 6-7-(left panels) and 12-(right panels) month-old *sh1* mice. Data are presented as mean±SEM, n indicates the number of eyes analyzed, the arrows point at the photopic ERG. No statistically significant differences were found between *sh1^+/−^* and *sh1^−/−^* mice. More details on the statistical analysis including specific statistical values can be found in the Statistical analysis paragraph of the Materials and Methods section. **B**. Representative scotopic ERG waves from one *sh1^+/−^* and one *sh1^−/−^* mouse at 12 months of age.
(TIF)

Figure S3 ***In vivo*** **retinal imaging in pigmented** ***sh1*** **mice.** Retinal optical coherence tomography (OCT) and scanning laser ophthalmoscopy (SLO) imaging were performed in *sh1^+/−^* control (**A–C**) and homozygous *sh1^−/−^* (**D–F**) mice at 12 months of age. The pictures depicted are representative of four *sh1^+/−^* mice (4 eyes analyzed) and two *sh1^−/−^* mice (2 eyes analyzed). The retinal layering was also compared to wild-type C57BL/6 mice (**G**). There were no fundus abnormalities visible in both *sh1^+/−^* control and *sh1^−/−^* affected mice in infrared mode (820 nm; **A**, **D**). The 3 black lines in these fundus images (**A**, **D**) indicate the positions from which representative OCT scans (**B, E**) are taken. A rectangle indicates the site from which the magnification shown in **c** and **f** originated, whereas an asterisk in the schematic orientation plot (**g**) marks the retinal origin of the OCT detail in the C57BL/6 mouse. D: dorsal; V: ventral; T: temporal; N: nasal; GC: ganglion cell; IPL: inner plexiform layer; INL: inner nuclear layer: OPL: outer plexiform layer; ONL: outer nuclear layer; OLM: outer limiting membrane; OS/IS: outer segment/inner segment border; RPE/ChC: retinal pigment epithelium/choriocapillaris.
(TIF)

Figure S4 **Southern blot analysis of DNA extracted from oversize AAV vectors.** Alkaline Southern blot analysis of DNA extracted from 3×10^10^ genome copies of oversize AAV vectors generated by using 3 different pAAV2.1 *cis*-plasmids containing large transgenes (from 7.7 to 8.9 Kb). The 3 panels represent 3 different exposure times of the same blot. Samples have been digested or not with DNase I. The 1 kb-ladder fragments size (Kb) is shown on the left. Arrows on the right point at DNA with molecular weight higher than 5 Kb.
(TIF)

Figure S5 **AAV-mediated expression of human Myo7a-HA** ***in vitro*** **.** Western blot analysis on HEK293 cell lysates following infection with AAV2/2-CMV-*hMYO7A-HA* (AAV-*hMYO7A-HA*) or AAV2/2-CMV-*EGFP* (AAV-*EGFP*) or transfection with pAAV2.1-CMV-*hMYO7A-HA* plasmid (p*-hMYO7A*). The human influenza hemagglutinin (HA) tag is located at hMyo7a C-terminus. The amount of protein loaded (µg) is showed. The arrow points at the hMyo7a-HA protein. The lysate from HEK293 cells transfected with the pAAV2.1-CMV-*hMYO7A* plasmid (p-*hMYO7A*) was used as positive control. HA: human influenza hemagglutinin tag.
(TIF)

Figure S6 **Western Blot analysis of murine Myo7a expression in the eyecups of** ***sh1^−/−^*** **and** ***sh1^+/−^*** **mice.** Western blot analysis of lysate eyecups from albino *sh^−/−^ and sh1^+/−^*mice using the anti-Myo7a antibody. The murine Myo7a protein was clearly detected in the eyecups of heterozygous *sh1^+/−^* mice (n = 2) but was not in the eyecups of *sh1^−/−^* mice (n = 2). The anti-Dysferlin antibody was used as loading control. One hundred µg of each eyecup lysate were loaded. The arrow points at the Myo7a protein.
(TIF)
