# Supplementary figures and images for: Myosin7a Deficiency Results in Reduced Retinal Activity Which Is Improved by Gene Therapy
Source: PLoS One. 2013 Aug 26;8(8):e72027. doi: 10.1371/journal.pone.0072027 (PMC3753344; doi:10.1371/journal.pone.0072027)

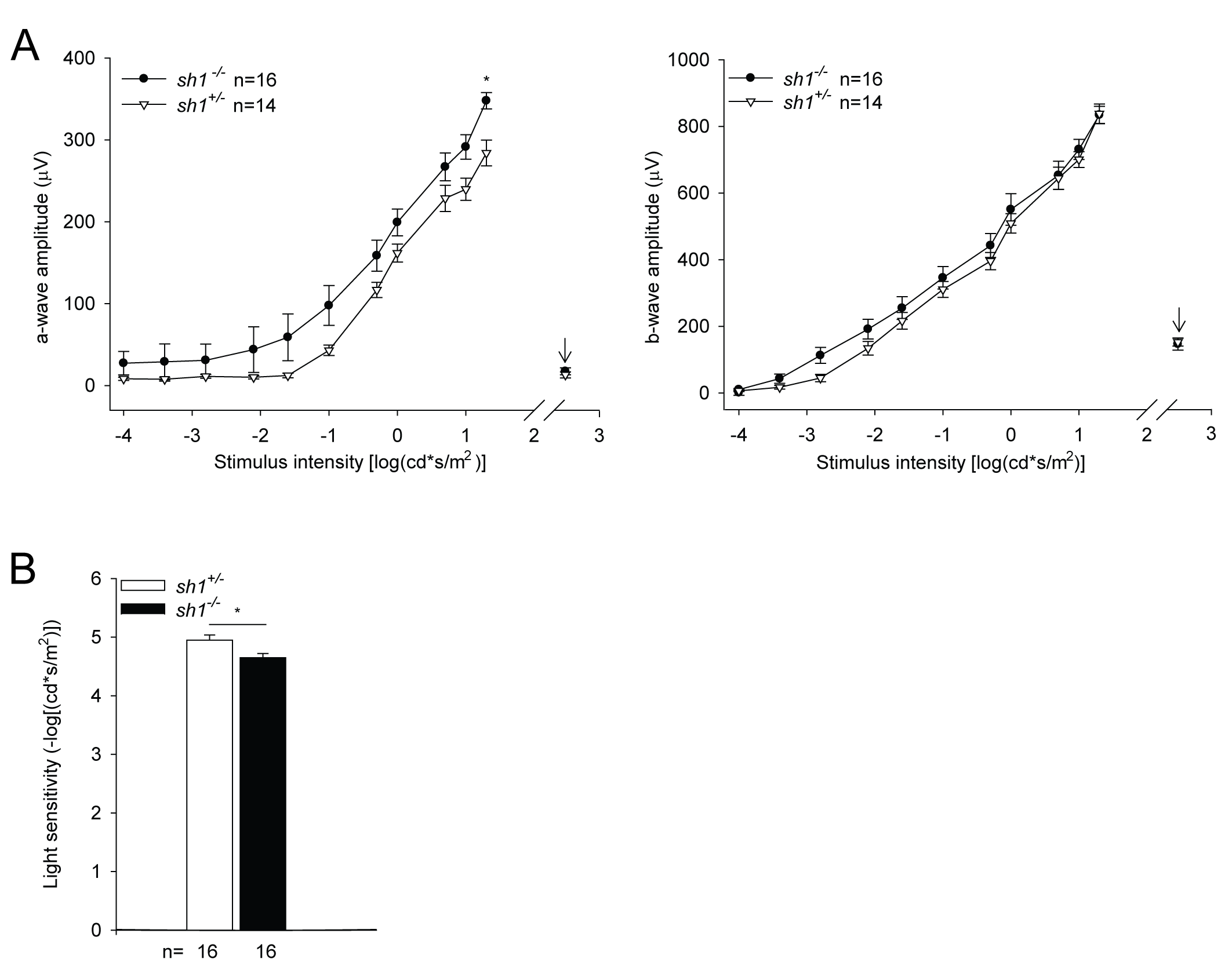

Supplement: Figure S1 — Ganzfeld electroretinograms (A) and retinal light sensitivity (B) of albino sh1 mice at 3 months of age. A–B. Data are presented as mean±SEM, n indicates the number of eyes analyzed, the arrows point at the photopic ERG. *p value<0.05. More details on the statistical analysis including specific statistical values can be found in the Statistical analysis paragraph of the Materials and Methods section. (TIF) [file pone.0072027.s001.tif]

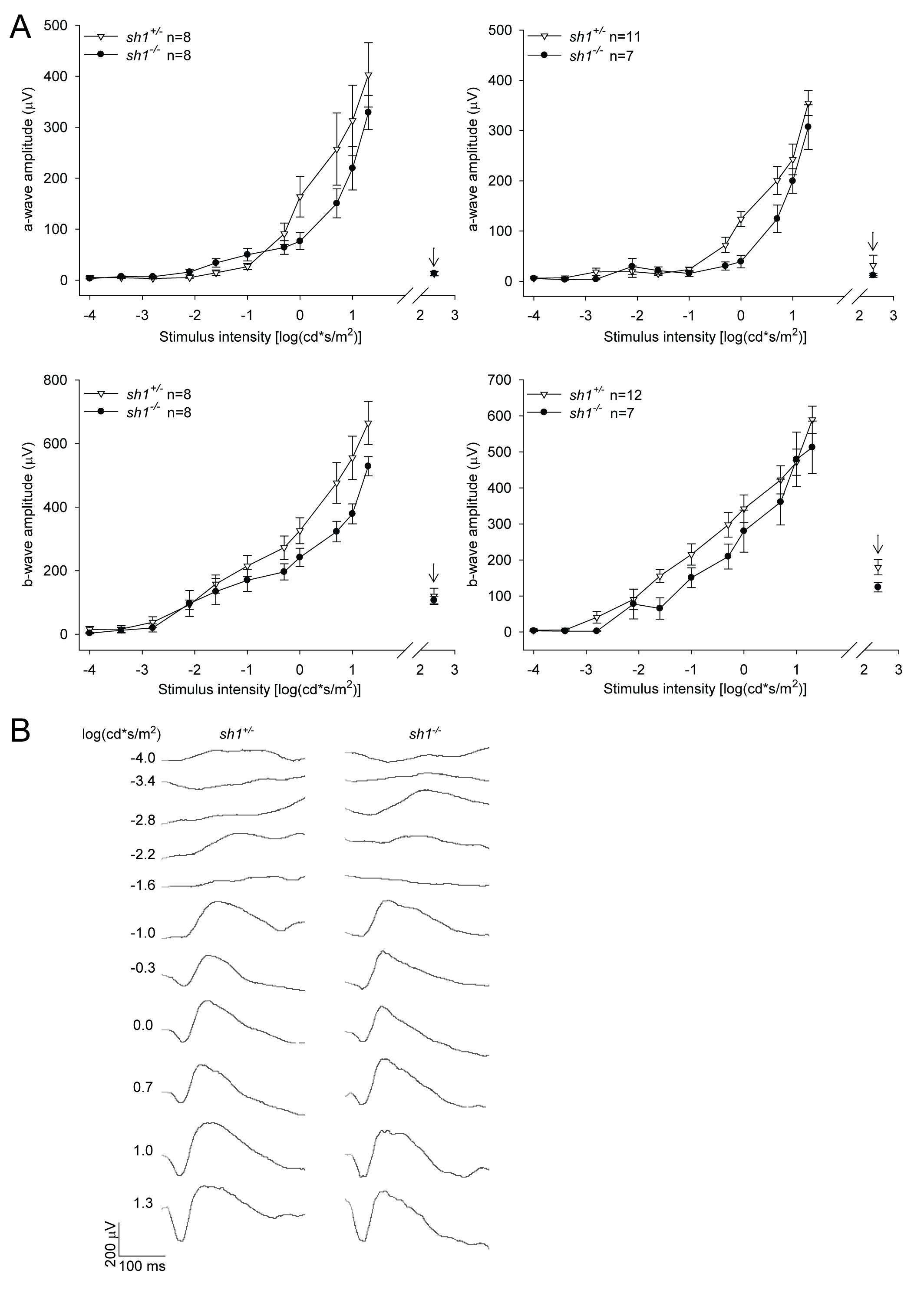

Supplement: Figure S2 — Ganzfeld electroretinograms in pigmented sh1 mice. A. Mean a- (top panels) and b-waves (bottom panels) from 6-7-(left panels) and 12-(right panels) month-old sh1 mice. Data are presented as mean±SEM, n indicates the number of eyes analyzed, the arrows point at the photopic ERG. No statistically significant differences were found between sh1+/− and sh1−/− mice. More details on the statistical analysis including specific statistical values can be found in the Statistical analysis paragraph of the Materials and Methods section. B. Representative scotopic ERG waves from one sh1+/− and one sh1−/− mouse at 12 months of age. (TIF) [file pone.0072027.s002.tif]

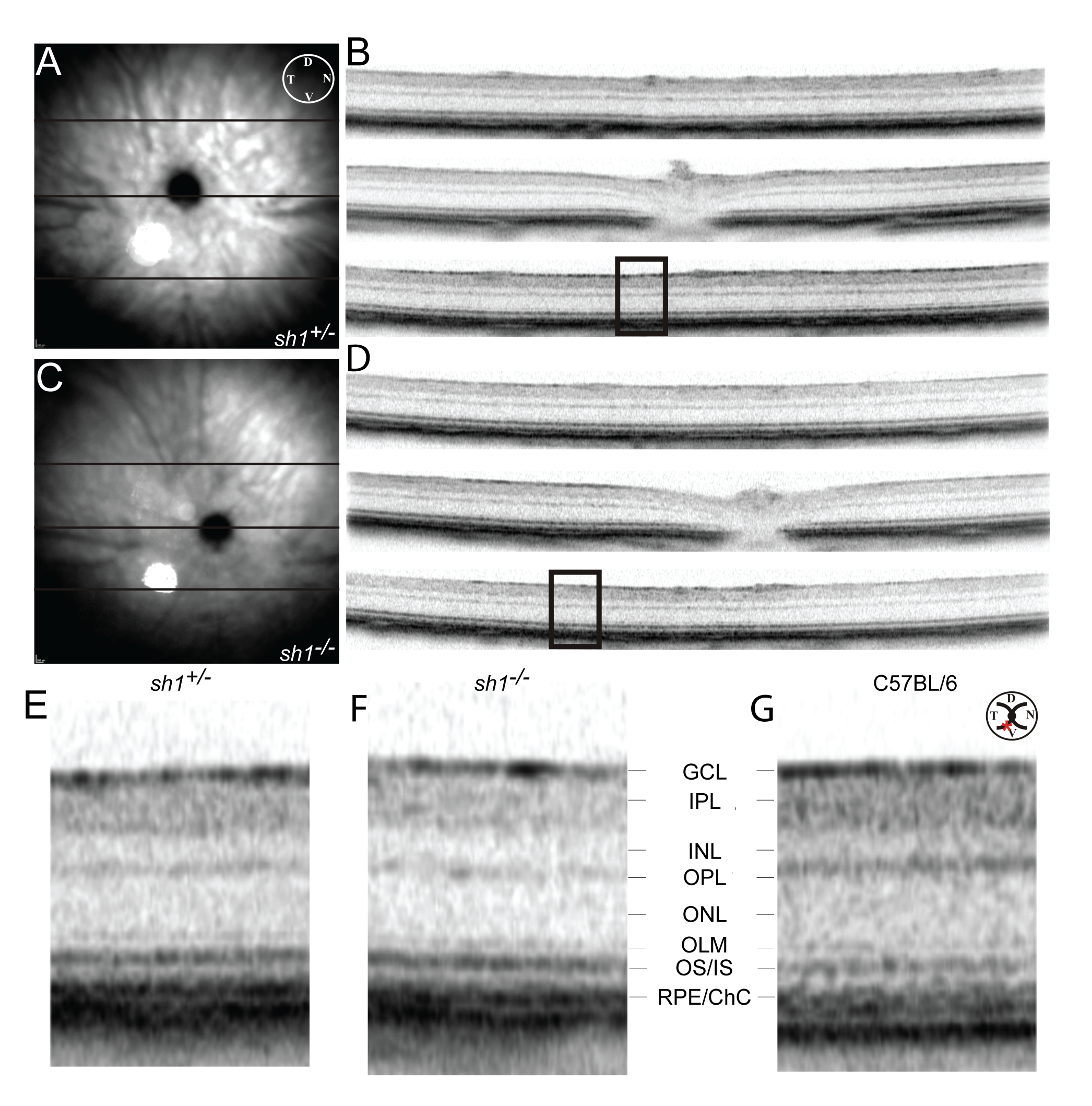

Supplement: Figure S3 — In vivo retinal imaging in pigmented sh1 mice. Retinal optical coherence tomography (OCT) and scanning laser ophthalmoscopy (SLO) imaging were performed in sh1+/− control (A–C) and homozygous sh1−/− (D–F) mice at 12 months of age. The pictures depicted are representative of four sh1+/− mice (4 eyes analyzed) and two sh1−/− mice (2 eyes analyzed). The retinal layering was also compared to wild-type C57BL/6 mice (G). There were no fundus abnormalities visible in both sh1+/− control and sh1−/− affected mice in infrared mode (820 nm; A, D). The 3 black lines in these fundus images (A, D) indicate the positions from which representative OCT scans (B, E) are taken. A rectangle indicates the site from which the magnification shown in c and f originated, whereas an asterisk in the schematic orientation plot (g) marks the retinal origin of the OCT detail in the C57BL/6 mouse. D: dorsal; V: ventral; T: temporal; N: nasal; GC: ganglion cell; IPL: inner plexiform layer; INL: inner nuclear layer: OPL: outer plexiform layer; ONL: outer nuclear layer; OLM: outer limiting membrane; OS/IS: outer segment/inner segment border; RPE/ChC: retinal pigment epithelium/choriocapillaris. (TIF) [file pone.0072027.s003.tif]

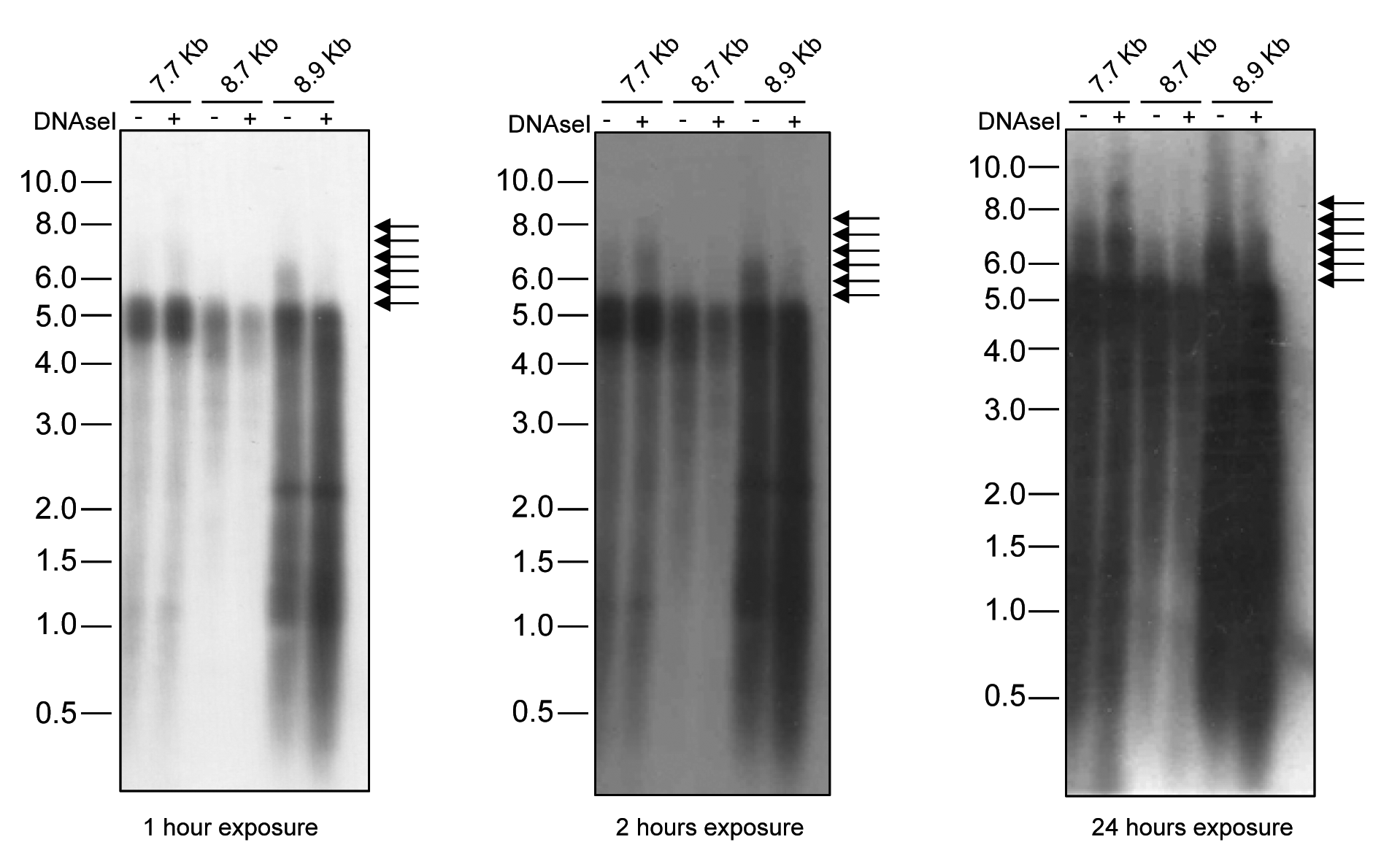

Supplement: Figure S4 — Southern blot analysis of DNA extracted from oversize AAV vectors. Alkaline Southern blot analysis of DNA extracted from 3×10^10^ genome copies of oversize AAV vectors generated by using 3 different pAAV2.1 cis-plasmids containing large transgenes (from 7.7 to 8.9 Kb). The 3 panels represent 3 different exposure times of the same blot. Samples have been digested or not with DNase I. The 1 kb-ladder fragments size (Kb) is shown on the left. Arrows on the right point at DNA with molecular weight higher than 5 Kb. (TIF) [file pone.0072027.s004.tif]

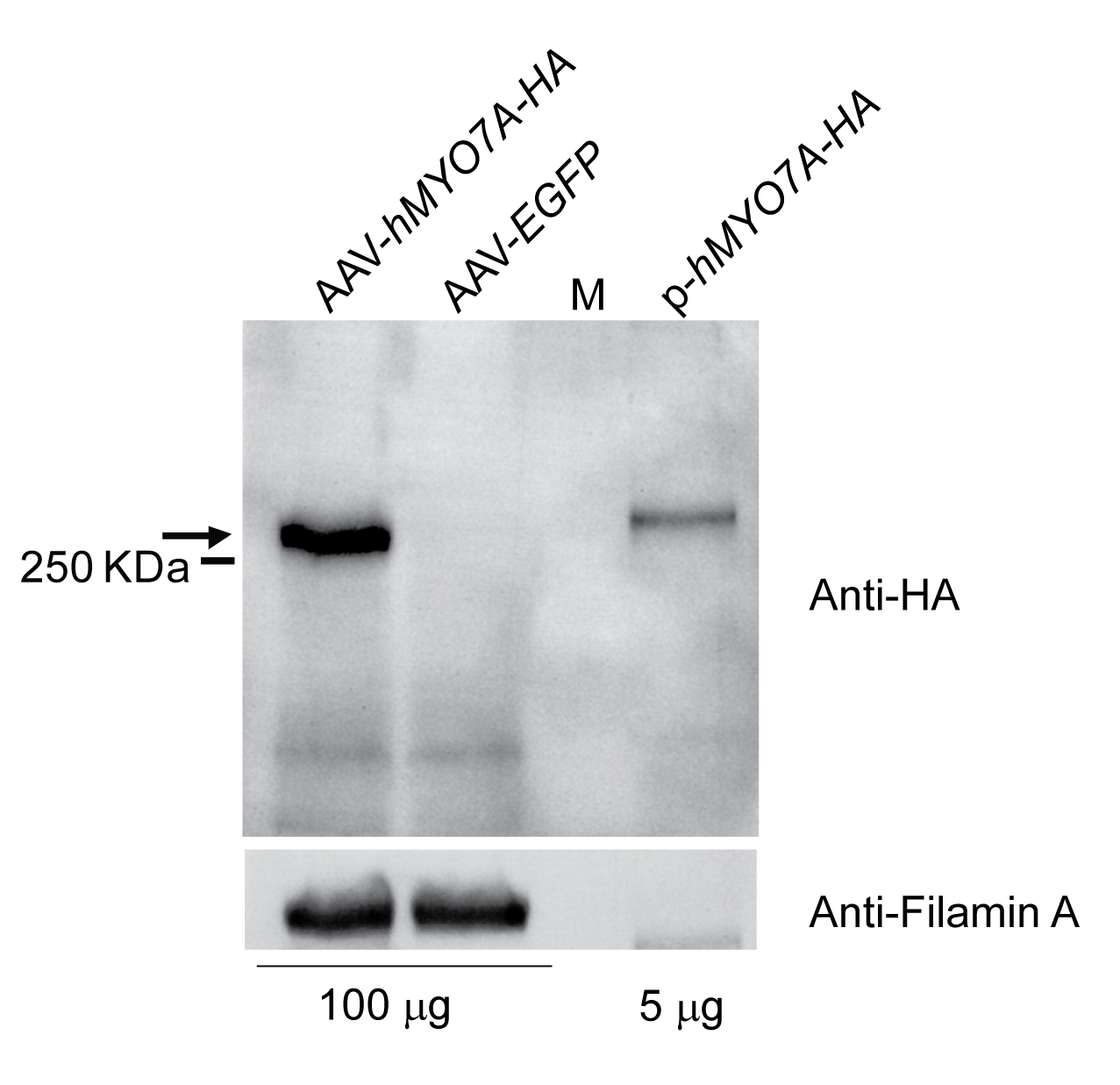

Supplement: Figure S5 — AAV-mediated expression of human Myo7a-HA in vitro . Western blot analysis on HEK293 cell lysates following infection with AAV2/2-CMV-hMYO7A-HA (AAV-hMYO7A-HA) or AAV2/2-CMV-EGFP (AAV-EGFP) or transfection with pAAV2.1-CMV-hMYO7A-HA plasmid (p-hMYO7A). The human influenza hemagglutinin (HA) tag is located at hMyo7a C-terminus. The amount of protein loaded (µg) is showed. The arrow points at the hMyo7a-HA protein. The lysate from HEK293 cells transfected with the pAAV2.1-CMV-hMYO7A plasmid (p-hMYO7A) was used as positive control. HA: human influenza hemagglutinin tag. (TIF) [file pone.0072027.s005.tif]

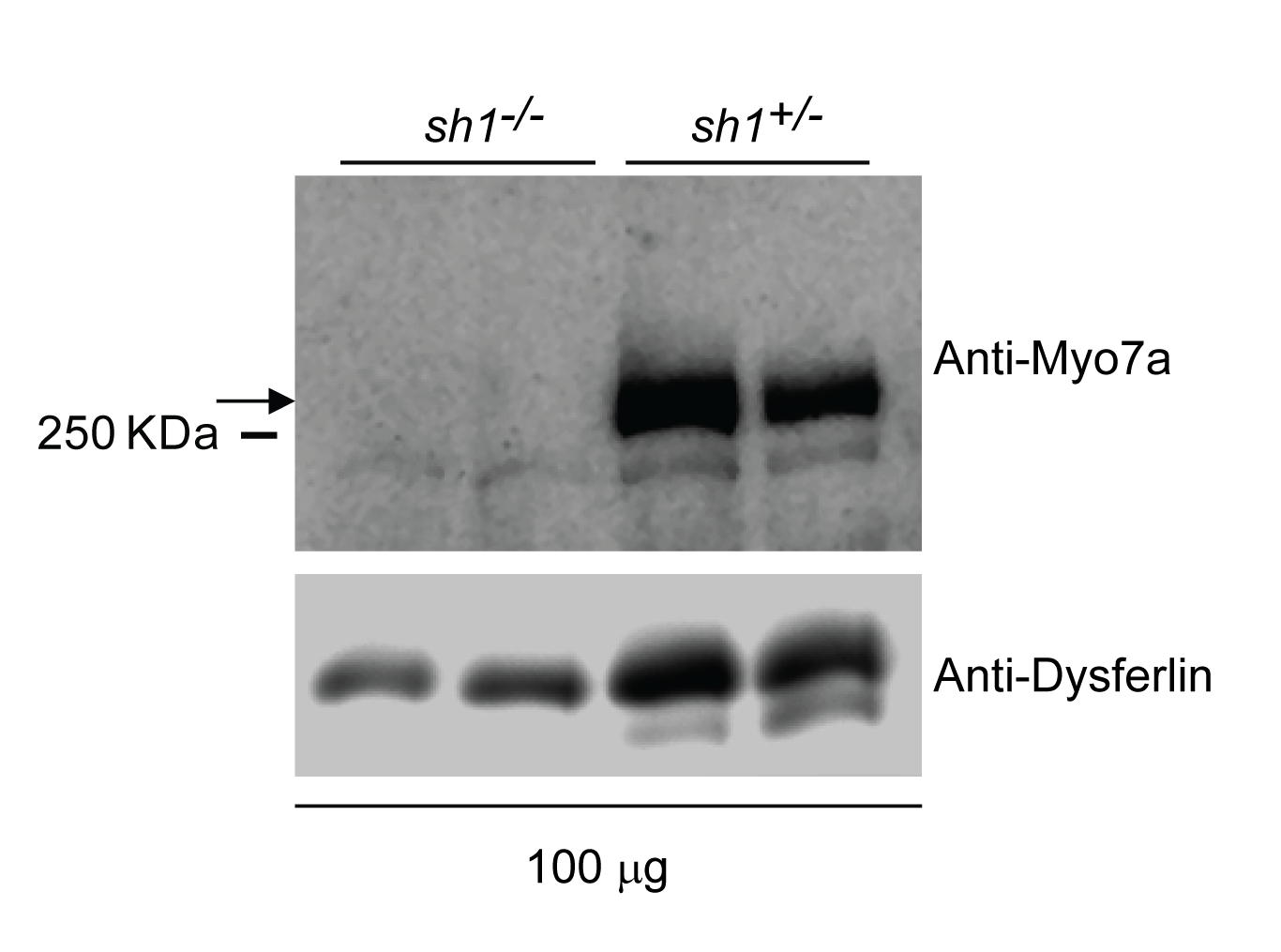

Supplement: Figure S6 — Western Blot analysis of murine Myo7a expression in the eyecups of sh1−/− and sh1+/− mice. Western blot analysis of lysate eyecups from albino sh−/− and sh1+/−mice using the anti-Myo7a antibody. The murine Myo7a protein was clearly detected in the eyecups of heterozygous sh1+/− mice (n = 2) but was not in the eyecups of sh1−/− mice (n = 2). The anti-Dysferlin antibody was used as loading control. One hundred µg of each eyecup lysate were loaded. The arrow points at the Myo7a protein. (TIF) [file pone.0072027.s006.tif]
